# Interaction between Brassica yellows virus silencing suppressor P0 and plant SKP1 facilitates stability of P0 *in vivo* against degradation by proteasome and autophagy pathways

**DOI:** 10.1111/nph.15702

**Published:** 2019-02-26

**Authors:** Yuanyuan Li, Qian Sun, Tianyu Zhao, Haiying Xiang, Xiaoyan Zhang, Zhanyu Wu, Cuiji Zhou, Xin Zhang, Ying Wang, Yongliang Zhang, Xianbing Wang, Dawei Li, Jialin Yu, Savithramma P. Dinesh‐Kumar, Chenggui Han

**Affiliations:** ^1^ State Key Laboratory for Agro‐Biotechnology and Ministry of Agriculture Key Laboratory of Pest Monitoring and Green Management China Agricultural University Beijing 100193 China; ^2^ State Key Laboratory of Agro‐Biotechnology and Ministry of Agriculture Key Laboratory of Soil Microbiology College of Biological Sciences China Agricultural University Beijing 100193 China; ^3^ Department of Plant Biology and The Genome Center College of Biological Sciences University of California, Davis Davis CA 95616 USA

**Keywords:** F‐box‐like motif, *Nicotiana benthamiana*, P0, polerovirus, protein stability, SKP1, suppression of RNA silencing

## Abstract

- P0 protein of some polerovirus members can target ARGONAUTE1 (AGO1) to suppress RNA silencing. Although P0 harbors an F‐box‐like motif reported to be essential for interaction with S phase kinase‐associated protein 1 (SKP1) and RNA silencing suppression, it is the autophagy pathway that was shown to contribute to AGO1 degradation. Therefore, the role of P0–SKP1 interaction in silencing suppression remains unclear.
- We conducted global mutagenesis and comparative functional analysis of P0 encoded by Brassica yellows virus (BrYV) (P0^Br^).
- We found that several residues within P0^Br^ are required for local and systemic silencing suppression activities. Remarkably, the F‐box‐like motif mutant of P0^Br^, which failed to interact with SKP1, is destabilized *in vivo*. Both the 26S proteasome system and autophagy pathway play a role in destabilization of the mutant protein. Furthermore, silencing of a *Nicotiana benthamiana SKP1* ortholog leads to the destabilization of P0^Br^. Genetic analyses indicated that the P0^Br^–SKP1 interaction is not directly required for silencing suppression activity of P0^Br^, but it facilitates stability of P0^Br^ to ensure efficient RNA silencing suppression. Consistent with these findings, efficient systemic infection of BrYV requires P0^Br^.
- Our results reveal a novel strategy used by BrYV for facilitating viral suppressors of RNA silencing stability against degradation by plant cells.

## Introduction

RNA silencing is an evolutionarily conserved regulator of gene expression and genome stability in eukaryotes. In plants, RNA silencing also functions as a potent antiviral defense mechanism (Ding & Voinnet, 2007; Pumplin & Voinnet, 2013). Antiviral RNA silencing is usually triggered by virus‐derived small interfering RNAs (vsiRNAs) generated from double‐stranded RNA (dsRNA) replication intermediates or intramolecular hairpin structures within the viral genomes by RNaseIII‐like enzymes called Dicer‐like proteins (DCLs) (Blevins *et al*., 2006; Deleris *et al*., 2006). One strand of the vsiRNA duplex then loads into an Argonaute (AGO)‐containing protein complex called RNA‐induced silencing complex (RISC) and can guide target viral mRNA slicing. Among 10 AGOs in *Arabidopsis*, AGO1 and AGO2 are two major players against RNA viruses (Morel *et al*., 2002; Qu *et al*., 2008; Vaucheret, 2008; Azevedo *et al*., 2010; Harvey *et al*., 2011; Jaubert *et al*., 2011; Wang *et al*., 2011; Carbonell *et al*., 2012; Dzianott *et al*., 2012; Zhang *et al*., 2012; Carbonell & Carrington, 2015; Garcia‐Ruiz *et al*., 2015). Viral mRNA cleavage then serves as a template for *de novo* dsRNA synthesis by RNA‐dependent RNA polymerase 6 (RDR6) and its cofactor, SUPPRESSOR OF GENE SILENCING 3 (SGS3). The synthesized dsRNAs are processed sequentially to produce ‘secondary’ vsiRNAs (Mourrain *et al*., 2000; Peragine *et al*., 2004; Vazquez *et al*., 2004; Schwach *et al*., 2005; Wang *et al*., 2011). Moreover, antiviral RNA silencing can spread to the surrounding cells or even to the distal organs (Voinnet *et al*., 1998; Dunoyer *et al*., 2005, 2010). The so‐called systemic RNA silencing represents the noncell‐autonomous nature of RNA silencing.

Plant viruses have evolved several proteins with considerable sequence and functional diversity to counteract the RNA silencing‐based antiviral defense, called viral suppressors of RNA silencing (VSRs). Various strategies are used by VSRs, including sequestration of double‐stranded vsiRNAs, inhibition of vsiRNA stabilization, and inactivation of the silencing effector proteins or competition with them (Diaz‐Pendon & Ding, 2008; Burgyan & Havelda, 2011; Incarbone & Dunoyer, 2013; Pumplin & Voinnet, 2013). The tombusvirus P19 protein binds to 21 nucleotide small interfering (siRNA) duplexes to prevent formation of the siRNA–AGO complex (Vargason *et al*., 2003; Ye *et al*., 2003). The P38 protein from *Turnip crinkle virus* (TCV) forms homodimers that bind AGO1 and possibly AGO2, and compromises AGO1 loading with siRNAs (Azevedo *et al*., 2010; Zhang *et al*., 2012). TGBp1 of *Plantago asiatica mosaic virus* (PlAMV) interacts with SGS3 and RDR6 to coaggregate and enwrap the SGS3‐RDR6 consortium (siRNA bodies) (Okano *et al*., 2014). Recently, investigations have shown that certain VSRs could mediate the degradation of various factors within the RNA silencing pathway. The polerovirus P0 protein has been shown to identify the degron in the DUF1785 domain of AGO1 and trigger degradation of AGO1 through the autophagy pathway (Baumberger *et al*., 2007; Bortolamiol *et al*., 2007; Csorba *et al*., 2010; Derrien *et al*., 2012, 2018) and the P25 of *Potato virus X* interacts with and mediates degradation of AGO1 through the proteasome pathway (Chiu *et al*., 2010). The VPg encoded by *Turnip mosaic virus* (TuMV) potyvirus mediates degradation of SGS3 via ubiquitination and autophagy pathways (Cheng & Wang, 2017). Because VSRs are pathogenicity factors or effectors that counteract antiviral silencing, they may be perceived and impaired by plants. However, this counter‐counter defense strategy used by plants and the final fate of VSRs during the virus–host arms race has not been fully explored.

P0 protein of *Turnip yellows virus* (TuYV), also known as *Beet western yellows virus* isolate FL1 (BWYV‐FL1), is the first VSR reported in the genus *polerovirus* (Pazhouhandeh *et al*., 2006). In recent several years, P0 proteins from various poleroviruses have been shown to function as VSRs (Pazhouhandeh *et al*., 2006; Mangwende *et al*., 2009; Csorba *et al*., 2010; Han *et al*., 2010; Kozlowska‐Makulska *et al*., 2010; Delfosse *et al*., 2014; Zhuo *et al*., 2014; Almasi *et al*., 2015; Cascardo *et al*., 2015; Chen *et al*., 2016). Several regions within P0 are essential for their silencing suppression activity, including a consensus F‐box‐like motif (LPXX(L/I)X_10–13_P) and the Phe/Trp (FW) residues within the C‐terminal consensus sequence ((K/R) IYGEDGX_3_FWR) (Pazhouhandeh *et al*., 2006; Bortolamiol *et al*., 2007; Mangwende *et al*., 2009; Han *et al*., 2010; Fusaro *et al*., 2012; Delfosse *et al*., 2014; Zhuo *et al*., 2014; Almasi *et al*., 2015; Chen *et al*., 2016). P0 proteins encoded by TuYV and a few other polerovirus members were reported to interact with S phase kinase‐associated protein 1 (SKP1), a member of the SKP1‐Cullin 1‐F‐box (SCF) E3 ubiquitin ligase complex, through the consensus F‐box‐like motif (Pazhouhandeh *et al*., 2006; Zhuo *et al*., 2014; Almasi *et al*., 2015). As P0 was reported to interact with AGO1 in the nucleus and trigger ubiquitylation and degradation of AGO1 in plants (Baumberger *et al*., 2007; Bortolamiol *et al*., 2007; Csorba *et al*., 2010; Fusaro *et al*., 2012), it was initially considered to hijack the host SCF machinery to destabilize AGO1. However, P0‐mediated AGO1 degradation is insensitive to inhibitors of the ubiquitin–proteasome system (Baumberger *et al*., 2007; Csorba *et al*., 2010), but it is blocked by inhibitor treatments or mutations impairing autophagy (Derrien *et al*., 2012). Therefore, P0 was proposed to inhibit RISC assembly by hijacking a normal host physiological process to promote selective autophagy of unloaded AGO1 (Derrien *et al*., 2012). In addition, P0 encoded by a *Potato leafroll virus* (PLRV) Inner Mongolian isolate (P0^PL‐IM^) triggers AGO1 degradation and suppresses RNA silencing without interaction with SKP1 (Zhuo *et al*., 2014). Hence, the role of the F‐box‐like motif and P0–SKP1 interaction in silencing suppression remains unclear.

Brassica yellows virus (BrYV) is a newly identified polerovirus infecting crucifer crops in China (Xiang *et al*., 2011). Sequence analysis has revealed that BrYV is closely related to but significantly different from TuYV, particularly the 5′‐terminal half of the genome, including P0 encoding sequence (Xiang *et al*., 2011; Zhang *et al*., 2014). It was previously shown that P0 of BrYV (P0^Br^) suppresses RNA silencing in *Nicotiana benthamiana* (Xiang & Han, 2011). Extensive targeted mutagenesis within P0^Br^ and comparative functional analysis indicate that distinct residues of P0^Br^ controls local and systemic RNA silencing suppression activities. More importantly, our investigation on the role of the P0^Br^–SKP1 interaction in RNA silencing suppression reveals a strategy facilitating the stability of BrYV VSR P0^Br^ to ensure its silencing suppression activity during virus infection. We note the low accumulation of the P0^Br^ F‐box‐like motif mutant and identify factors responsible for it. Virus‐induced gene silencing (VIGS) further demonstrates that knockdown of an *N. benthamiana SKP1* ortholog (*NbSKP1*), the host factor interacting with P0 via its F‐box‐like motif, destabilizes P0 protein. Genetic evidence also shows that, rather than directly playing a role in silencing suppression, the interaction between P0^Br^ and SKP1 seems to ensure silencing suppression activity through facilitating the stability of P0^Br^. In addition, our results indicate an important role for P0^Br^ in BrYV systemic infection.

## Materials and Methods

### Plant material and growth conditions

*Nicotiana* *benthamiana*, a green fluorescent protein (GFP) transgenic *N. benthamiana* 16c line, and a P0^Br^‐6Myc transgenic *N. benthamiana* line were germinated from seeds and maintained at 24°C with a 13 h (*c*. 75 μmol m^−2^ s) daylight regimen.

### Genes and plasmid constructs

All the primers used in this study are listed in Supporting Information Table S1.

Construction of pGD‐P0^Br^‐3Flag was described previously (Sun *et al*., 2018). For transient expression, cDNA of P38 was amplified from TCV (Qu *et al*., 2003) and cloned into *Hin*dIII and *Bam*HI sites of pGD (Goodin *et al*., 2002) to produce pGD‐P38^TCV^. Mutants of P0^Br^ were produced by inverse PCR amplification (Geier & Modrich, 1979; Sambrook & Russell, 2001) and cloned into pGD‐3FLAG to produce desired mutants. The 5′ fragment of BrYV infectious cDNA clones containing various P0 mutants was generated in the pTBrA001‐3430 by inverse PCR (Zhang *et al*., 2015). The resulting plasmids were digested with *Stu*I and *Afl*II, and ligated together with the *Afl*II‐ and *Bgl*II‐digested fragment from pTBrA3251‐Bgl3R into the *Stu*I and *Bam*HI sites of pCB301‐2x35S‐MCS‐HDV_RZ_‐NOS to produce the desired mutants (Yao *et al*., 2011; Zhang *et al*., 2015). For the construct used in generation of the transgenic *N. benthamiana* plants, BrYV P0 was cloned into pGD‐6Myc, a modified version of vector pGD that contains a C‐terminal 6Myc tag. A fragment of 6Myc‐tagged P0 was then cloned into pER8 to produce pER8‐P0^Br^‐6Myc (Zuo *et al*., 2000). For yeast‐two hybrid assays, P0^Br^ and its derivative mutants were cloned into the *Nde*I and *Bam*HI sites of pGBKT7. Construction of pGAD‐NbSKP1 was described previously (Wang Q. *et al*., 2013). For transient expression of GFP‐tagged NbSKP1 and FLAG‐tagged GUS, the *NbSKP1* gene and *GUS* gene were amplified from pGAD‐NbSKP1 and P31GUS, respectively (Wang Q. *et al*., 2013; Wu *et al*., 2014), and cloned into *Xho*I and *Apa*I sites of pGDGm (a modified version of pGD which allows the production of C‐terminal GFP‐fused protein) or pGD‐3FLAG to produce pGD‐NbSKP1‐GFP or pGD‐GUS‐3FLAG. Full‐length NbSKP1 without start or stop codon was cloned into pTRV2 to produce pTRV2‐*NbSKP1* (Liu *et al*., 2002).

### Transient coexpression assay and observation of RNA silencing

Plasmids were transformed into the *Agrobacterium tumefaciens* strain EHA105 or C58CI using the freeze–thaw method (Holsters *et al*., 1978). Co‐infiltration assays were performed as previously described (Zhuo *et al*., 2014).

GFP fluorescence in the infiltrated leaves and systemic leaves was illuminated under a BLAK‐RAY non‐UV semiconductor inspection lamp (B‐100AP/R; UVP Inc., Upland, CA, USA) and photographed using a digital camera (CoolPix 4500; Nikon, Tokyo, Japan) with a yellow filter (Kodak Wratten gelatin filter, no. 15) at 2 and 14 d post‐infiltration (dpi), respectively. The number of systemically silenced 16c plants was measured for each treatment, and the silencing ratio was calculated from plants tested in four different experiments. All experiments were repeated three times.

### RNA extraction, semiquantitative reverse transcriptase PCR and RNA gel blot analysis

Plant total RNA used for RNA and sRNA gel blots was extracted using Trizol Reagent (Invitrogen) according to the manufacturer's protocol. Isolated RNA was reverse transcribed using oligo (dT) primer HC51118TR and Moloney murine leukemia virus (M‐MLV) reverse transcriptase (Promega). Semiquantitative reverse transcriptase PCR (RT‐PCR) was performed as described previously (Wang Y. *et al*., 2013).

Total RNA was separated in a 1.5% denaturing gel and then blotted to Hybond‐N+ membrane (Amersham Pharmacia Biotech). Templates of probes were amplified by PCR with respective primers and labeled with [α‐^32^P] dCTP using the Prime‐a‐Gene Labeling System (Promega) in hybridization buffer (Sigma‐Aldrich) at 65°C for 16 h. The signals were detected by exposing the membrane to X‐ray film with the chemical fluorescent substrate. Signal intensity was quantified using imagequant tl software (GE Healthcare, Little Chalfont, UK).

### Protein extraction and western blotting

As described previously (Sun *et al*., 2018), total proteins were extracted from infiltrated patches of *N. benthamiana* leaves using ×2 sodium dodecyl sulfate (SDS) sample buffer (100 mM Tris (pH 6.8), 4% SDS, 20% glycerol and 0.2% bromophenol blue) containing 10% β‐mercaptoethanol. Total yeast proteins were extracted as described (Kushnirov, 2000). Proteins were separated on 12.5% or 6% (for detection of 6Myc‐AtAGO1) polyacrylamide gels, and transferred onto polyvinylidene fluoride membranes. The membranes were blotted with the FLAG antibody (Sigma‐Aldrich), c‐Myc antibody (Sigma‐Aldrich), or polyclonal antiserum against GFP or NbSKP1, and subsequently detected by goat anti‐rabbit horseradish peroxidase‐conjugated antibody (Bio‐Rad) followed by chemiluminescence detection (GE Healthcare). To quantify the protein, coomassie brilliant blue R250 was used (0.1% in 50% methanol : 12% acetic acid) to stain the gel overnight with gentle shaking.

### Generation of P0^Br^‐6Myc transgenic *N*. *benthamiana* plants

The pER8‐P0^Br^‐6Myc plasmid was introduced into *Agrobacterium* strain EHA105, followed by leaf disk transformation of *N*. *benthamiana* plants as described previously (Horsch *et al*., 1989). After cultivation and regeneration of leaf explants, genomic DNA was isolated with a standard CTAB method (Doyle & Doyle, 1987), and PCR analysis was performed to screen the positive transgenic plants (Table S1). Leaves of transgenic plants were treated with 100 mM β‐estradiol 2 d before sample collection to induce expression of 6Myc‐tagged P0 protein.

### GAL4 yeast two‐hybrid assay

Yeast two‐hybrid (Y2H) experiments were performed with the Matchmaker GAL4 Two‐Hybrid System 3 (Clontech, Palo Alto, CA, USA) as previously described (Sun *et al*., 2018).

### *In vivo* co‐immunoprecipitation

Co‐immunoprecipitation (co‐IP) was performed as previously reported with minor modifications (Win *et al*., 2011). Agro‐infiltrated leaf tissue from *N. benthamiana* was ground under liquid nitrogen and homogenized in 2 ml g^−1^ extraction buffer (10% glycerol, 25 mM Tris‐HCl (pH 7.5), 1 mM EDTA, 150 mM NaCl, 2% (w/v) polyvinylpolypyrrolidone, 10 mM dithiothreitol, ×1 protease inhibitor cocktail (Sigma‐Aldrich), 0.1% Triton X‐100 (Sigma‐Aldrich)). After centrifugation at 3000 ***g*** for 10 min at 4°C and filtration with a 0.45 mm filter, the clarified lysate was incubated with 4% BSA preblocked anti‐FLAG M2 agarose beads (Sigma‐Aldrich) for 3 h and the complex was washed five times with immunoprecipitation buffer (10% glycerol, 25 mM Tris (pH 7.5), 1 mM EDTA, 150 mM NaCl, 0.1% Triton X‐100). The immunoprecipitates were denatured and subjected to immunoblotting using corresponding antibodies.

### VIGS assay

For the VIGS assay (Liu *et al*., 2002), the constructed pTRV2 vectors mentioned above were introduced into *A. tumefaciens* strain GV3101. *Agrobacterium* harboring TRV1 or TRV2 derivative vectors were mixed at a 1 : 1 ratio and infiltrated into the leaves of 4‐wk‐old *N. benthamiana* plants.

### Inhibitor treatment

MG132 (Merck Calbiochem, Billerica, MA, USA) was used at the final concentration of 100 μM. E‐64d (Selleck Chemicals, Houston, TX, USA) was used at a final concentration of 50 μM. For inhibitor treatment, MG132 or E‐64d was infiltrated into *N. benthamiana* leaves for 10 h before harvesting. Dimethylsulfoxide (DMSO) was used as a solvent control.

### *Agrobacterium*‐mediated inoculation of virus

Constructed cDNA clones of BrYV and its derivative mutants based on pCB301‐2x35S‐MCS‐HDV_RZ_‐NOS were transformed into *A. tumefaciens* strain C58CI. The *Agrobacterium* harboring cDNA clones were infiltrated into 4‐ to 5‐wk‐old *N. benthamiana* plants.

## Results

### Global mutagenesis of P0^Br^ determines several residues essential for P0^Br^‐mediated suppression of RNA silencing

**Figure 1:**
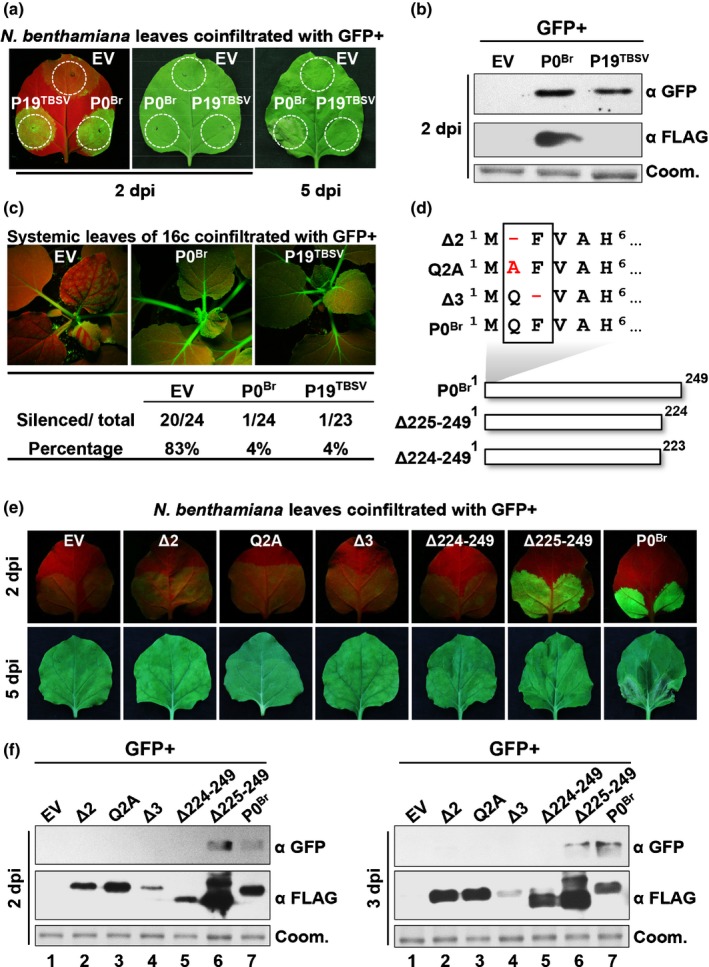
Deletion mutagenesis of Brassica yellows virus P0 protein (P0^Br^) determines regions essential for P0^Br^‐mediated suppression of RNA silencing and cell death. (a) Suppression of local RNA silencing and induction of cell death by P0^Br^. GFP was transiently coexpressed by *Agrobacterium*‐infiltration in *Nicotiana benthamiana* leaves together with 3FLAG‐tagged P0^Br^ and photographs were taken under long‐wavelength UV light at 2 d post‐infiltration (dpi) and under visible light at 2 and 5 dpi. Empty vector (EV) and P19 protein encoded by *Tomato bushy stunt virus* (P19^TBSV^) were used as negative and positive controls. (b) Immunoblot analysis of GFP and P0^Br^ in co‐infiltrated patches of *N. benthamiana* leaves from (a). GFP and 3FLAG‐tagged P0^Br^ were detected by western blot analyses with GFP polyclonal antiserum (α GFP) and FLAG monoclonal antibody (α FLAG), respectively. Coomassie stain (Coom.) of total protein is shown to indicate equal loading. (c) Suppression of systemic RNA silencing by P0^Br^. GFP was transiently coexpressed in leaves of GFP transgenic *N. benthamiana* (line 16c) together with 3FLAG‐tagged P0^Br^ and photographs of the upper leaves were taken under long‐wavelength UV light at 14 dpi. Empty vector (EV) and P19^TBSV^ were used as negative and positive controls. The number of plants showing systemic silencing was calculated and compared with the total number of co‐infiltrated plants tested in four independent experiments. (d) Schematic representation of amino acids deleted (−) or mutated (red) in the N‐terminus of P0^Br^ (upper panel) and C‐terminal truncation mutants (lower panel). Numbers correspond to the amino acid positions within the P0^Br^ sequence. (e) Suppression of local RNA silencing and induction of cell death by P0^Br^ mutants. GFP was transiently coexpressed in *N. benthamiana* leaves together with 3FLAG‐tagged P0^Br^ or its mutants Δ2, Δ3, Q2A, Δ224–249 and Δ224–249. Photographs were taken under long‐wavelength UV light at 2 dpi and under visible light at 5 dpi. Empty vector (EV) was used as a negative control. (f) Immunoblot analysis of GFP and 3FLAG‐tagged P0^Br^ or its mutants in co‐infiltrated patches of *N. benthamiana leaves* from (e). GFP and 3FLAG‐tagged proteins were detected by western blot analyses with GFP polyclonal antiserum (α GFP) and FLAG monoclonal antibody (α FLAG), respectively. Coomassie stain (Coom.) of total proteins is shown to indicate equal loading.

We revealed previously that BrYV P0 is a strong viral suppressor of RNA silencing (Xiang & Han, 2011). Here, we confirmed this result using the widely used *Agrobacterium*‐mediated transient coexpression assay in *N. benthamiana* (Fig. 1a,b) (Voinnet *et al*., 2000; Johansen & Carrington, 2001). Meanwhile, P0^Br^ induces cell death in infiltrated *N. benthamiana* leaves at 5 dpi (Fig. 1a, right panel). To analyze the ability of P0^Br^ to suppress the long‐distance spread of RNA silencing (systemic silencing), an *Agrobacterium*‐mediated coexpression assay was performed in the GFP transgenic *N. benthamiana* line 16c (Ruiz *et al*., 1998). In this assay, expression of GFP in the lower leaves leads to silencing of GFP in the newly emerging upper leaves of 16c plants. In plants co‐infiltrated with empty vector (EV), *c*. 80% of the plants exhibited systemic RNA silencing at 14 dpi (Fig. 1c). Conversely, systemic RNA silencing was suppressed in the upper leaves of plants co‐infiltrated with P0^Br^ or the positive control P19 protein of *Tomato bushy stunt virus* (P19^TBSV^) (Fig. 1c). These results indicate that BrYV P0 is a strong VSR which suppresses both local and systemic RNA silencing.

To determine the regions of P0^Br^ essential for VSR activity and induction of cell death, a series of truncation or deletion mutations were generated (Fig. 1d). In addition, each residue in P0^Br^ except Ala residues was substituted with Ala by alanine‐scanning mutagenesis. P0^Br^ or its mutant derivatives were transiently coexpressed with GFP in the leaves of wild‐type or 16c transgenic *N. benthamiana* plants. A single amino acid deletion at position 2 or 3 in the N‐terminus of P0^Br^ (Δ2 or Δ3) or Ala substitution of Gln2 (Q2A), Try61 (Y61A), Try96 (Y96A) or Try211 (Y211A) abolished RNA silencing suppression and cell death induction (Fig. 1e and Table 1). Deletion of 25 amino acids in the C‐terminus of P0^Br^ (Δ225–249) or Ala substitution of Leu184 (L184A) or His206 (H206A) abolished systemic RNA silencing suppression and cell death induction without affecting local RNA silencing suppression (Fig. 1e and Table 1). Interestingly, deletion of one additional amino acid at the C‐terminus (Δ224–249) abolished both VSR and cell death induction function of P0^Br^ (Table 1 and Fig. 1e). Western blot analyses showed that GFP protein accumulation (Fig. 1f) was consistent with GFP fluorescence levels (Fig. 1e). These results indicated that the N‐terminus of P0^Br^ and three Try residues (Try61, Try96 and Try211) are important for both cell death and RNA silencing suppression function of P0^Br^, but the C‐terminus residues 225–249 and two residues (Leu184 and His206) are important for systemic RNA silencing suppression and cell death induction but not for local RNA silencing suppression activities.

**Table 1:** Summary of the biological activities of Brassica yellows virus P0 mutants

Mutation type	Name	Local RSS^a^	Systemic RSS^b^	Cell death^c^	AGO1 degradation^d^	NbSKP1 interaction^e^
Truncation mutant	Δ3	−	−	−	−	−
	Δ2	sr	−	−	sr	Reduced
	Δ225–249	+	−	−	+	nd
	Δ224–249	−	−	−	−	nd
Conserved sequence mutant	LP (L63A/P64A)	−	+	+	−	−
	LPK (L63A/P64A/K44A)	+	−	Reduced	+	−
	F219R	−	−	−	nd	nd
Alanine substitution mutant	Q2A	−	−	−	−	+
	Y61A	−	−	−	−	−
	Y96A	−	−	−	−	−
	Y211A	−	−	−	−	−
	H108A	+	−	sr	+	nd
	F149A	+	−	sr	+	nd
	W153A	+	nd	sr	+	nd
	L184A	+	−	−	+	+
	R186A	+	nd	sr	+	nd
	D191A	+	nd	sr	+	nd
	H195A	+	−	sr	+	nd
	H206A	+	−	−	nd	nd
	I210A	+	nd	sr	+	nd
Other substitution mutant	A88F	−	−	−	−	−
	Y61D	−	−	−	nd	−

nd, no data; sr, significantly reduced.

a Results of local RNA silencing suppression (RSS) in co‐infiltrated *Nicotiana benthamiana* plants are scored as either positive (+) or negative (−) as determined by GFP fluorescence visual detection under a long‐wave UV lamp, western blot and/or RNA blot assays at 2 d post‐infiltration (dpi).

b Results of systemic RNA silencing suppression in co‐infiltrated GFP transgenic *N. benthamiana* 16c line plants are scored as either positive (+) or negative (−) as measured by systemic silencing ratio of assayed plants at 14 dpi.

c Cell death is marked as either positive (+) or negative (−) as determined by visual observation of infiltrated *N. benthamiana* plants at 5 dpi.

d The results of AGO1 degradation in infiltrated *N. benthamiana* plants are marked as either positive (+) or negative (−) as detected by Western blot analyses at 2 dpi.

e The results of protein interaction with NbSKP1 are marked as either positive (+) or negative (−) as detected by a yeast two‐hybrid assay and/or co‐immunoprecipitation assay.

**Figure 2:**
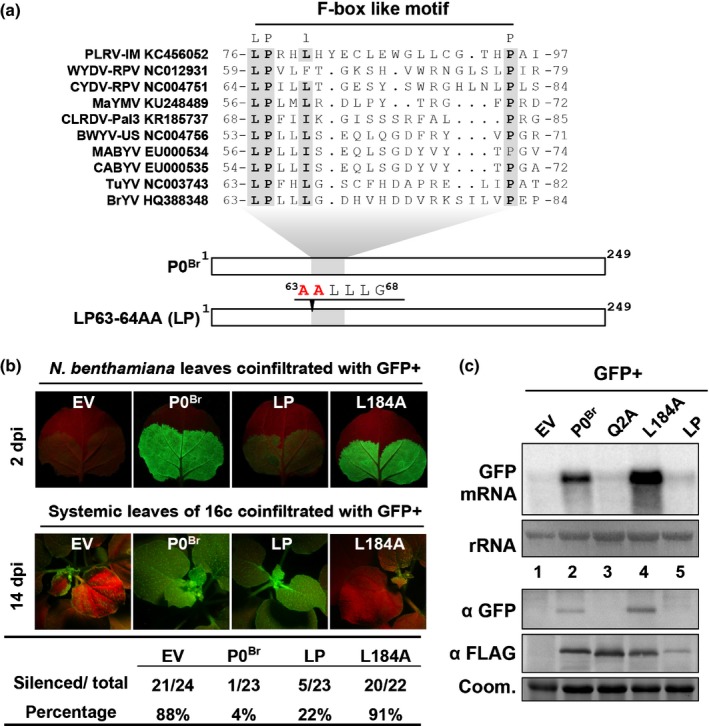
The F‐box‐like motif and the leucine at position 184 in P0^Br^ are essential for local and systemic silencing suppression, respectively. (a) Alignment of the P0 sequence from 10 different viruses of the genus *polerovirus* and schematic representation of mutations in the F‐box‐like motif of P0^Br^. P0^Br^ and its region containing the F‐box‐like motif are represented by white and gray boxes, respectively. Landmark residues of the F‐box‐like motif are highlight in gray. Amino acid sequences harboring mutated amino acids (depicted in red) are presented above the open reading frames (ORFs) at the mutated positions indicated by black arrowheads. Numbers correspond to the amino acid positions within the P0^Br^ sequence. (b) Suppression of RNA silencing by P0^Br^ mutants. GFP was transiently coexpressed by *Agrobacterium*‐infiltration in *Nicotiana benthamiana* or GFP transgenic *N. benthamiana* (line 16c) leaves together with 3FLAG‐tagged P0^Br^ (P0^Br^) or its mutants LP and L184A. Empty vector (EV) was used as a negative control. Fluorescence images of *N. benthamiana* infiltrated leaves and 16c upper leaves were taken under long‐wavelength UV light at 2 d post‐infiltration (dpi) and 14 dpi, respectively. The number of plants showing systemic silencing was calculated and compared with the total number of co‐infiltrated plants tested in four independent experiments. (c) Northern blot analysis of GFP mRNA and immunoblot analysis of GFP and 3FLAG‐tagged P0^Br^ or its mutants in co‐infiltrated patches of *N. benthamiana* leaves at 2 dpi. mRNAs of GFP were hybridized with a random primed 3′ untranslated region (UTR) *‐*specific probe. Methylene blue staining of rRNAs after northern transfer was used as loading control for high‐molecular‐weight RNA blots (rRNA). GFP and 3FLAG‐tagged proteins were detected by western blot analyses with GFP polyclonal antiserum (α GFP) and FLAG monoclonal antibody (α FLAG), respectively. Coomassie stain (Coom.) of total proteins is shown to indicate equal loading.

A conserved F‐box‐like motif and an FWR sequence are present in P0^Br^: 63‐LPLLLGDHVHDDVRKSILVP‐82 (Fig. 2a) and 209‐KIYGEDGFISFWRIA‐223 (Fig. S1a). Ala substitutions of Leu63 and Pro64 (LP63‐64AA, hereafter abbreviated as LP) in the F‐box‐like motif and Arg substitution of Phe219 (F219R) in the FWR motif in P0^Br^ also abolished its ability to suppress local RNA silencing (Figs 2b,c, S1b), indicating the requirement of these motifs for local silencing suppression activity of P0^Br^. Surprisingly, the LP mutant is still able to suppress systemic silencing compared to F219R (Figs 2b,c, S1b).

### P0^Br^ F‐box mutant LP63‐64AA is destabilized and degraded by the 26S proteasome and autophagy pathways

**Figure 3:**
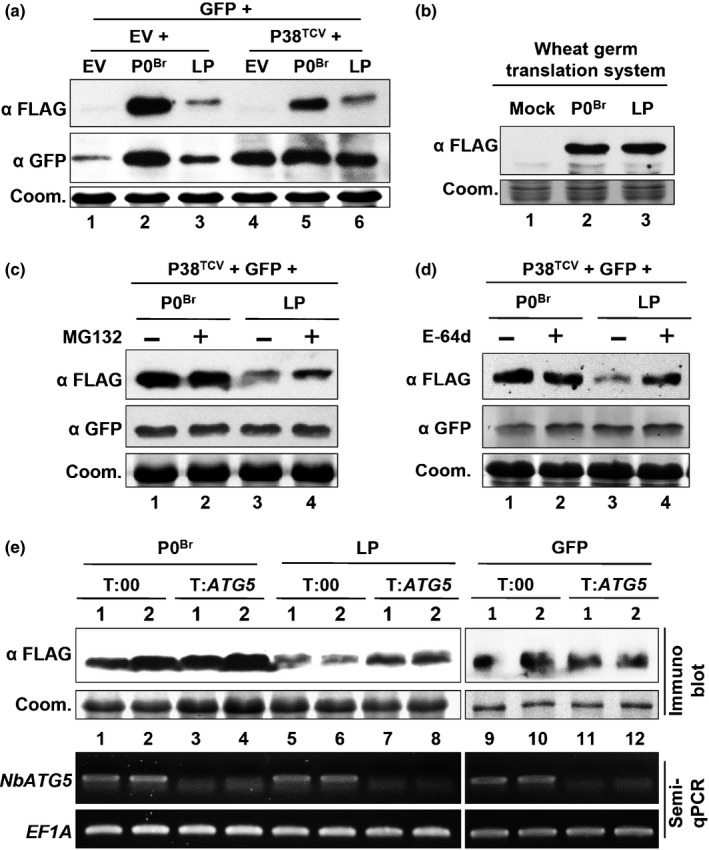
Mutations in the F‐box‐like motif destabilizes P0^Br^. (a) Effect of P38^TCV^ on the protein accumulation of P0^Br^ and its mutant LP in *Nicotiana benthamiana*. In the presence or absence of P38^TCV^, GFP was transiently coexpressed in *N. benthamiana* leaves together with 3FLAG‐tagged P0^Br^ or its mutant LP. Empty vector (EV) was used as a negative control. Total proteins were extracted from co‐infiltrated patches at 2 d post‐infiltration (dpi). GFP and 3FLAG‐tagged proteins were analyzed by western blotting with GFP polyclonal antiserum (α GFP) and FLAG monoclonal antibody (α FLAG), respectively. Coomassie stain (Coom.) of total proteins is shown to indicate equal loading. (b) Accumulation of *in vitro* translated P0^Br^ and its mutant LP. Ten micrograms of *in vitro* transcribed capped mRNA was used as template. The translational products of *P0* ^*Br*^ *‐3FLAG* mRNA (P0^Br^) and its mutant *LP‐3FLAG* mRNA (LP) were analyzed by western blotting with FLAG monoclonal antibody (α FLAG). A reaction without mRNA served as a negative control (Mock). Coomassie stain (Coom.) of total proteins is shown to indicate equal loading. (c, d) Effect of different proteasome inhibitors on the protein accumulation of P0^Br^ and its mutant LP in *N. benthamiana*. 3FLAG‐tagged P0^Br^ or its mutant LP was transiently expressed in *N. benthamiana* together with GFP and P38^TCV^. Total protein was extracted from infiltrated patches at 2 dpi. MG132 at 100 µM (c) or E‐64d at 50 μM (d) was infiltrated into *N. benthamiana* leaves for 12 h before harvesting (+); DMSO treatment was used as a solvent control (−). Accumulation of GFP and 3FLAG‐tagged P0^Br^ or LP were analyzed by western blotting with GFP polyclonal antiserum (α GFP) and FLAG monoclonal antibody (α FLAG), respectively. Coomassie stain (Coom.) of total protein is shown to indicate equal loading. (e) Effect of *NbATG5* knockdown on the protein stabilization of P0^Br^ and its mutant LP in *N. benthamiana*. A TRV‐based VIGS system was used to silence *NbATG5* (T:*ATG5*). TRV:00 empty vector was used as a control (T:00). At 20 dpi, the silenced upper leaves were infiltrated with *Agrobacterium* containing construct encoding 3FLAG‐tagged P0^Br^ or its mutant LP. Accumulation of 3FLAG‐tagged proteins was detected by western blot analyses with FLAG monoclonal antibody (α FLAG). Coomassie stain (Coom.) of total proteins is shown to indicate equal loading. *NbATG5* mRNA was analyzed by semiquantitative reverse transcription (RT)‐PCR. *EF1A* was used as an internal control.

We noted low accumulation of LP mutant protein compared to wild‐type P0^Br^ or its silencing‐suppression‐deficient mutant Q2A (Fig. 2c). This could be due to RNA silencing, low translation efficiency and/or destabilization of protein. Therefore, we transiently coexpressed the LP mutant with P38^TCV^ VSR that is predicted to suppress RNA silencing and rescue protein accumulation. However, the LP level was still low compared to P0^Br^ in the presence of P38^TCV^ VSR (Fig. 3a, top panel, lanes 5 and 6). By contrast, GFP accumulated to the same level in different samples in the presence of P38^TCV^ (Fig. 3a, middle panel, lanes 4–6). These results indicate that deficiency in silencing suppression activity may not be the only reason for the low level of the LP mutant. We then compared the translation efficiency of the LP mutant with that of P0^Br^ by performing *in vitro* translation experiments in wheat germ extracts using T7‐derived transcripts. LP showed equivalent translation efficiency to that of P0^Br^ in the wheat germ translation system (Fig. 3b). These results suggest that low accumulation of LP may be due to destabilization of protein *in vivo*.

There are two major fundamentally different mechanisms in cells by which proteins are degraded, autophagy and the proteasome (Klionsky & Emr, 2000; Smalle & Vierstra, 2004). To investigate pathways that contribute to the protein destabilization of LP, we tested different inhibitors including MG132 (a peptide aldehyde that inhibits 20S proteasome activity by covalently binding to the active site of the β subunits) and E‐64d (a membrane‐permeable cysteine protease inhibitor that can inhibit the degradation of autophagic cargo inside autolysosomes by blocking the activity of a subset of lysosomal hydrolases). P0^Br^ or LP was transiently coexpressed in *N. benthamiana* leaves together with P38^TCV^, and the infiltrated patches were treated with different inhibitors before harvest. In the presence of P38^TCV^, GFP accumulated to the same level in the samples with or without treatment of MG132 (Fig. 3c, middle panel, lanes 1–4) or E‐64d (Fig. 3d, middle panel, lanes 1–4). The MG132 or E‐64d treatment did not cause obvious change in the accumulation level of P0^Br^ protein (Fig. 3c,d, top panel, lanes 1 and 2). By contrast, LP accumulated to a higher level in the presence of MG132 (Fig. 3c, top panel, lanes 3 and 4), although the level was still unable to reach to that of P0^Br^. Interestingly, accumulation of LP increased significantly in the presence of E‐64d to the level of P0^Br^ (Fig. 3d, top panel, lanes 3 and 4). These results suggest that destabilization of LP is likely to be a consequence of protein degradation occurring through both the 26S proteasome pathway and the autophagy pathway.

ATG5 is an important player in activating autophagy, and silencing of *NbATG5* has been shown to suppress autophagy in *N. benthamiana* (Wang Y. *et al*., 2013, 2015; Klionsky *et al*., 2016; Haxim *et al*., 2017). To further confirm the inhibitor results, we silenced the *N. benthamiana ATG5* (*NbATG5*) gene using a TRV‐based VIGS system (Fig. S2) (Wang Y. *et al*., 2013). At 20 dpi, P0^Br^ or LP was transiently coexpressed in the upper leaves of TRV‐infected *N. benthamiana* plants together with P38^TCV^, and samples were collected and assayed 2 d later. Semiquantitative RT‐PCR revealed that *NbATG5* mRNA accumulation was significantly reduced in TRV:*NbATG5* infected plants compared with TRV:00 infected plants (Fig. 3e). Silencing of *NbATG5* did not affect the protein level of GFP (Fig. 3e, top panel, lanes 9–12), but slightly increased the protein level of LP (Fig. 3e, top panel, lanes 5–8). This result suggests that autophagy may play a role in destabilization of LP.

### Silencing of *NbSKP1* results in a significant reduction of BrYV P0 protein *in vivo*

**Figure 4:**
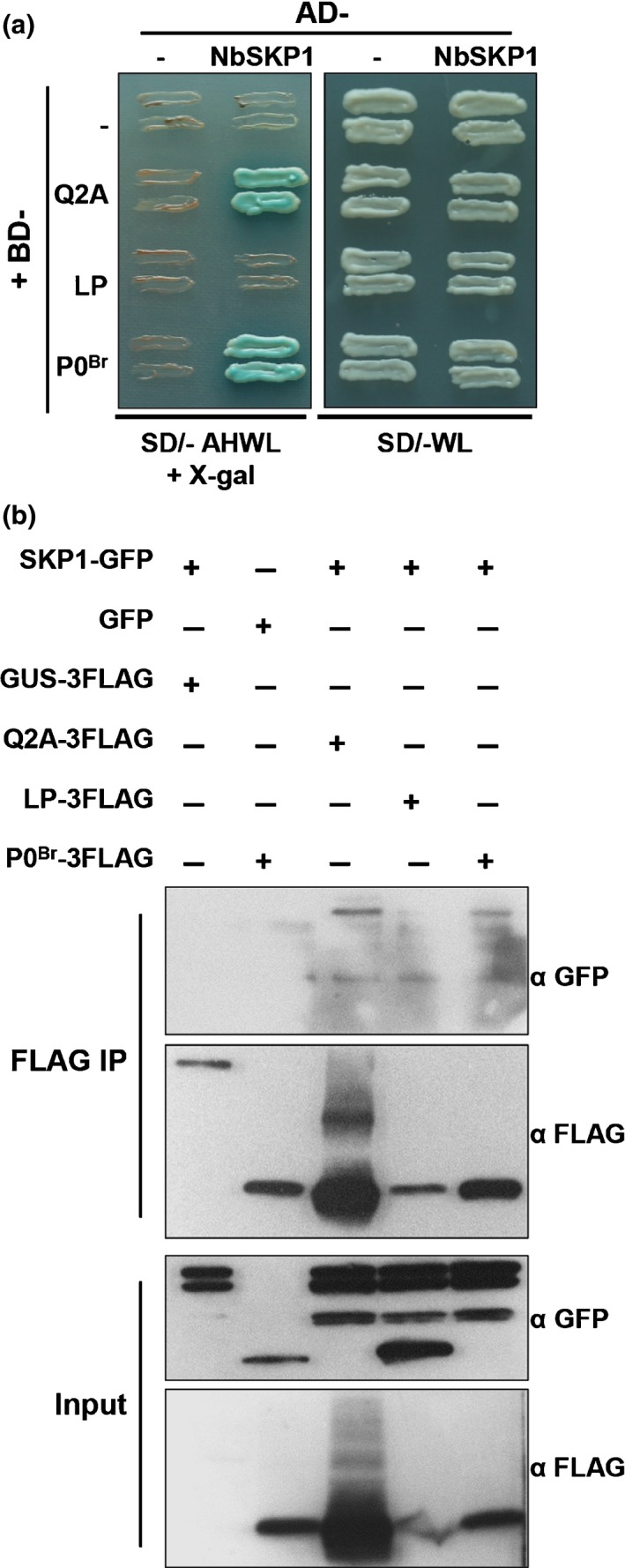
F‐box‐like motif of P0^Br^ is required for the P0^Br^–NbSKP1 interaction. (a) Analysis of interactions between NbSKP1 and P0^Br^ or its derivative mutants in a yeast two‐hybrid system. P0^Br^ or its derivative mutants Q2A and LP were cloned into bait vector pGBKT7 and then transferred into yeast Y187. NbSKP1 was cloned into prey vector pGADT7 and transferred into yeast AH109. Successful yeast mating resulted in vigorous growth on synthetic dropout (SD) media lacking Trp and Leu (SD/−WL). Interaction was indicated by yeast growth and blue color (representing LacZ activity) on SD media containing X‐gal and lacking Ade, Trp, Leu and His (SD/− AHWL + X‐gal). (b) Co‐immunoprecipitation analyses of P0^Br^ and NbSKP1 proteins in *Nicotiana benthamiana* leaves. 3FLAG‐tagged P0^Br^ or its derivative mutants Q2A and LP were transiently coexpressed with NbSKP1‐GFP in *N. benthamiana*. 3FLAG‐tagged GUS was used as a negative control. Protein complexes were immunoprecipitated using ANTI‐FLAG M2 Affinity Gel. GFP‐tagged proteins or 3FLAG‐tagged proteins in input and immunoprecipitation fractions (Input and IP) were immunoblotted using GFP polyclonal antiserum (α GFP) or FLAG monoclonal antibody (α FLAG).

To determine if P0^Br^ or its F‐box‐like motif mutants interact with SKP1, we performed a Y2H assay. P0^Br^ and its mutants (Q2A and LP) were fused to the GAL4 DNA binding domain (BD) as bait and *N. benthamiana* SKP1 (NbSKP1, GenBank accession no. AF494084.1) ortholog was fused to the GAL4 activation domain (AD) as prey. P0^Br^ and Q2A interacted with NbSKP1 as indicated by yeast growth and the presence of a blue color (LacZ activity) on SD media lacking Ade, His, Trp and Leu (SD/−AHLW) with X‐gal (5‐bromo‐4‐chloro‐3‐indolyl‐β‐d‐galactoside) (Fig. 4a, left panel). By contrast, the F‐box‐like motif mutant LP failed to interact with NbSKP1 (Fig. 4a), although fusion protein was expressed (Fig. S4, left panels). To further confirm these results, co‐IP analysis was performed. GFP‐tagged NbSKP1 (NbSKP1‐GFP) and FLAG‐tagged P0^Br^ or its mutants were coexpressed in *N. benthamiana* leaves through agroinfiltration. Protein extracts were immunoprecipitated with anti‐FLAG conjugated beads followed by western blot analyses with anti‐GFP and anti‐FLAG antibodies. NbSKP1‐GFP co‐immunoprecipitated with P0^Br^‐3FLAG and Q2A‐3FLAG (Fig. 4b; lanes 4 and 2) but not with LP‐3FLAG (Fig. 4b, lane 3) or GUS‐3FLAG control (Fig. 4b, lane 1). These results confirmed that the F‐box‐like motif is required for P0–SKP1 interaction.

**Figure 5:**
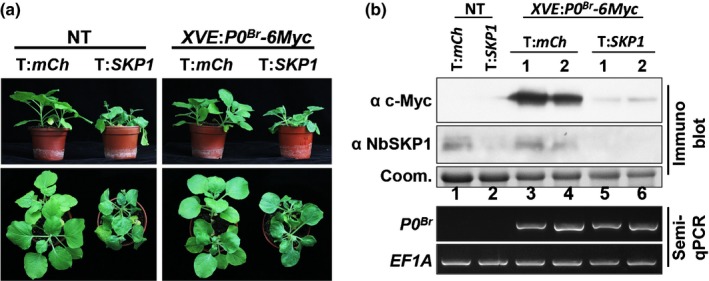
Silencing of *NbSKP1* impairs the protein accumulation of BrYV P0 protein in *Nicotiana benthamiana*. (a) Symptoms of the *N. benthamiana* plants infected with TRV:*mCherry* or TRV:*NbSKP1*. A TRV‐based VIGS system was used to silence *NbSKP1* (T:*SKP1*) in nontransgenic (NT) or *XVE::P0* ^*Br*^ *‐6Myc* transgenic *N. benthamiana*. TRV:*mCherry* empty vector was used as a control (T:*mCh*). Photographs of plants were taken at 20 d post‐infiltration (dpi). (b) Silencing of *NbSKP1* impairs the protein accumulation of BrYV P0 protein *in vivo*. Estradiol (100 μM) was applied to the *NbSKP1*‐silenced upper leaves at 19 dpi to induce expression of P0^Br^‐6Myc in transgenic plants. Leaves were harvested 2 d after estradiol treatment for protein and RNA extraction. Accumulation of 6Myc‐tagged P0^Br^ proteins and NbSKP1 were analyzed by western blotting with c‐Myc monoclonal antibody (α c‐Myc) and NbSKP1 polyclonal antiserum (α NbSKP1), respectively. Coomassie stains of total proteins are shown to indicate equal loading (Coom.). Expression of *P0* ^*Br*^ *‐6Myc* mRNA was analyzed by reverse transcription (RT) semiquantitative‐PCR (semi‐qPCR). *EF1A* was used as an internal control.

To determine the role of SKP1 in stabilization of P0^Br^, we silenced *N. benthamiana SKP1* orthologs using TRV‐based VIGS. We generated P0^Br^ inducible *N. benthamiana* transgenic lines, in which the expression of P0^Br^‐6Myc transgene driven by the XVE promoter can be induced upon treatment with β‐estradiol (Fig. S5) (Zuo *et al*., 2000). Expression of P0^Br^‐6Myc was induced upon β‐estradiol treatment in upper leaves of TRV:*NbSKP1* infected plants and TRV:*mCherry* infected control plants (Fig. 5a), and samples were collected and assayed 2 d later. Accumulation of NbSKP1 protein was significantly reduced in TRV:*NbSKP1* infected plants compared with TRV:*mCherry* infected plants (Fig. 5b). Silencing of *NbSKP1* caused a significant decrease in P0^Br^‐6Myc protein accumulation without an effect on its mRNA level (Fig. 5b, lanes 3–6). These results suggest that the presence of SKP1 facilitates stability of P0^Br^ protein *in vivo*.

### The P0^Br^ F‐box‐like motif mutant LPK44A maintains RNA silencing suppression activity without interacting with SKP1

**Figure 6:**
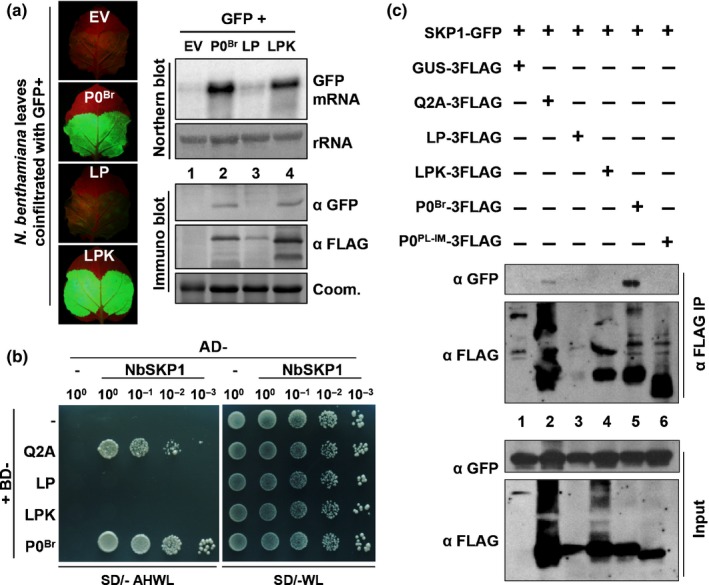
RNA silencing suppression by P0^Br^ does not require the P0^Br^–NbSKP1 interaction. (a) Effects of alanine substitution of lysine at position 44 (K44A) on protein stabilization and silencing suppression activity of the P0^Br^ mutant LP. GFP was transiently coexpressed in *Nicotiana benthamiana* leaves together with empty vector (EV) or proteins indicated above the lanes. GFP mRNA was detected by northern blot analysis using a random primed 3′ untranslated region (UTR)‐specific probe. Methylene blue staining of rRNAs after northern transfer was used as a loading control (rRNA). GFP and 3FLAG‐tagged P0^Br^ or its derivative mutants were detected by western blot analyses with GFP polyclonal antiserum (α GFP) and FLAG monoclonal antibody (α FLAG), respectively. Coomassie stain (Coom.) of total protein is shown to indicate equal loading. Fluorescence images of *N. benthamiana* infiltrated leaves were taken under long‐wavelength UV light at 2 d post‐infiltration (dpi). (b) Analysis of interactions between NbSKP1 and P0^Br^ or its derivative mutants in the yeast two‐hybrid system. P0 or its derivative mutants were cloned into bait vector pGBKT7 and then transferred into yeast Y187. NbSKP1 was cloned into prey vector pGADT7 and transferred into yeast AH109. Successful yeast mating resulted in vigorous growth on synthetic dropout (SD) media lacking Trp and Leu (SD/–WL). Interaction was indicated by yeast growth and blue color (representing LacZ activity) on SD media lacking Ade, Trp, Leu and His (SD/– AHWL). (c) Co‐immunoprecipitation analyses of P0^Br^ and NbSKP1 in *N. benthamiana* leaves. 3FLAG‐tagged P0^Br^ or its derivative mutants Q2A, LP and LPK were transiently coexpressed with NbSKP1‐GFP in *N. benthamiana*. 3FLAG‐tagged GUS and P0 encoded by a *Potato leafroll virus* Inner Mongolian isolate (P0^PL^ ^‐^ ^IM^) were used as a negative control. Protein complexes were immunoprecipitated using ANTI‐FLAG M2 Affinity Gel. GFP‐ or FLAG‐tagged proteins in input and immunoprecipitation fractions (Input and IP) were immunoblotted using GFP polyclonal antiserum (α GFP) and FLAG monoclonal antibody (α FLAG).

To identify a second site mutation within LP that could stabilize the protein expression, we generated and analyzed various Ala‐scanning LP mutants in *N. benthamiana* plants. We found that Ala substitution of Lys at position 44 (K44A) in LP (LP63‐64AA/K44A, hereafter abbreviated LPK) rescued the protein accumulation (Fig. 6a, left panels). Surprisingly, this LPK mutant suppressed local RNA silencing compared to LP in the GFP transient coexpression assay (Fig. 6a, right panels). Western blot analyses showed that GFP protein accumulation (Fig. 6a, left panels) was consistent with GFP fluorescence levels (Fig. 6a, right panels). These results further established that lack of local RNA silencing by LP is due to protein degradation.

To determine whether the additional K44A mutation rescues the interaction between LP and SKP1, we performed a Y2H assay. P0^Br^ and its mutant Q2A both interacted with NbSKP1 as described above (Figs 4a, 6b). By contrast, F‐box‐like motif mutants LP and LPK failed to interact with NbSKP1 (Fig. 6b), although fusion proteins were expressed (Fig. S4, right panels). To further confirm these results, co‐IP analyses were performed. Consistent with the Y2H results, NbSKP1‐GFP co‐immunoprecipitated with P0^Br^‐3FLAG and its mutant Q2A‐3FLAG (Fig. 6c; lanes 5 and 2) but not with mutant LP‐3FLAG, LPK‐3FLAG (Fig. 6c, lanes 3 and 4), wild‐type P0^PL‐IM^‐3FLAG, or GUS‐3FLAG control (Fig. 6c, lane 1). These results show that although the P0^BrA^ Q2A mutant can interact with NbSKP1 it fails to suppress local silencing (Fig. 2). Furthermore, the LPK mutant promotes suppression of silencing but fails to interact with NbSKP1. In conclusion, these novel results indicate that interaction with NbSKP1 is neither essential nor sufficient for P0^Br^ to suppress RNA silencing.

### AGO1 destabilization triggered by P0^Br^ is correlated with the suppression of local RNA silencing

**Figure 7:**
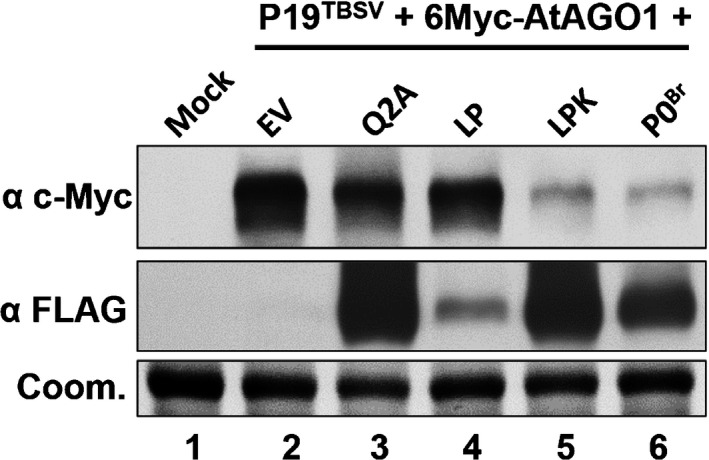
Effects on AGO1 protein stability by P0^Br^ and its mutants. 6Myc‐tagged AtAGO1 was transiently coexpressed by *Agrobacterium*‐infiltration in *Nicotiana benthamiana* leaves together with empty vector (EV), 3FLAG‐tagged P0^Br^ or its mutants Q2A, LP and LPK in the presence of P19^TBSV^. Total protein was extracted from co‐infiltrated patches at 2 d post‐infiltration (dpi). Accumulation of 6Myc‐tagged AtAGO1 and 3FLAG‐tagged P0^Br^ or its mutants was analyzed by western blotting with c‐Myc monoclonal antibody (α c‐Myc) and FLAG monoclonal antibody (α FLAG), respectively. Coomassie stain of total proteins is shown to indicate equal loading (Coom.).

Polerovirus P0 proteins have been reported to target AGO1, a key component of the RISC, for degradation through the autophagy pathway. Furthermore, AGO1 degradation was shown to be essential for RNA silencing suppression of P0 (Baumberger *et al*., 2007; Bortolamiol *et al*., 2007; Derrien *et al*., 2012). To investigate the ability of P0^Br^ and its mutants (Q2A, LP, LPK) to target AGO1 for degradation, 6Myc‐tagged *Arabidopsis* AGO1 (6Myc‐AtAGO1) and 3FLAG‐tagged P0^Br^ or its mutants were coexpressed in the leaves of *N. benthamiana* as previously described (Baumberger *et al*., 2007; Duan *et al*., 2012). P19^TBSV^ VSR was added in the assay to ensure expression of 6Myc‐AtAGO1 and P0^Br^ mutants. In the absence of P0 proteins, 6Myc‐AtAGO1 accumulated to a high level at 2 dpi (Fig. 7, lane 2). By contrast, 6Myc‐AtAGO1 accumulated to a significantly lower level in the presence of the P0^Br^ and LPK mutant (Fig. 7, lanes 6 and 5). In the presence of P0^Br^ mutants defective in local silencing suppression (LP and Q2A), 6Myc‐AtAGO1 accumulated to a similar level as that of the empty vector (pGD) (Fig. 7, lanes 3 and 4). Similar to results from a previous report (Derrien *et al*., 2012), AGO1 degradation mediated by P0^Br^ could be blocked by the E‐64d inhibitor, indicating a similar role of the autophagy pathway in this degradation process (Fig. S3). These results suggested that the local RNA silencing suppression function of the P0^Br^ and LPK mutant is correlated with destabilization of AGO1 triggered by them.

### P0^Br^ is required for efficient systemic infection of BrYV

**Figure 8:**
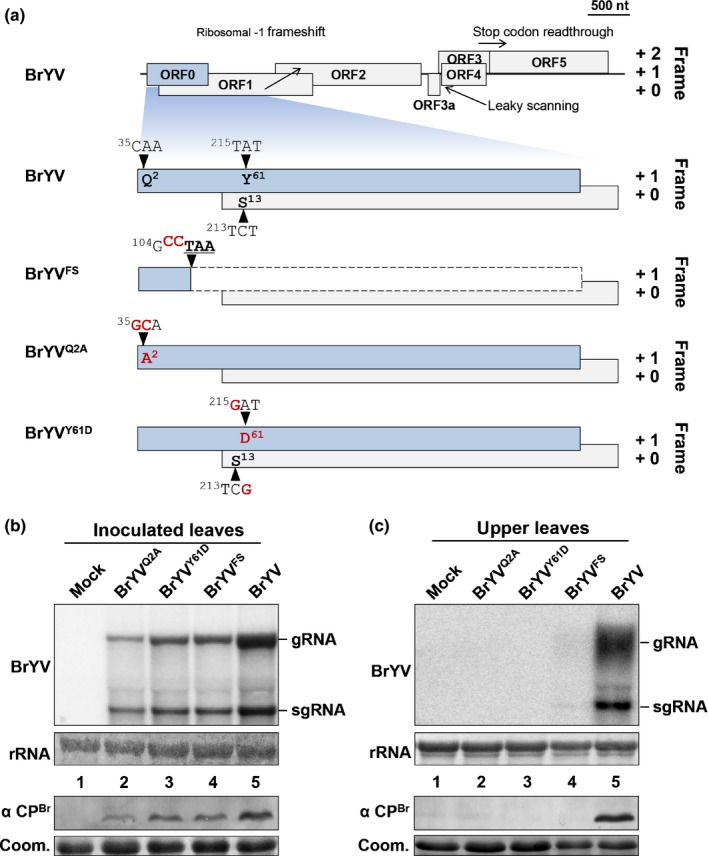
Accumulation of BrYV and its derivative mutants in *Nicotiana benthamiana* plants. (a) Schematic representation of the BrYV genome structure and the mutation strategies. Blue box and gray boxes represent different open reading frames (ORFs) of BrYV; thick black line indicates viral cDNA. Ribosomal frameshift, leaky scanning and stop codon readthrough sites are indicated by arrows. Sequences harboring nucleotides and amino acids (depicted in red) are presented above the ORFs at the mutated positions indicated by black arrowheads. The small blue box followed by a dashed box represents the prematurely terminated translation product of ORF0, which is a result of a frameshift caused by the insertion of two C residues at position 105 of the BrYV genome RNA. (b, c) Accumulation of BrYV and its derivative mutants in *N. benthamiana* plants was analyzed by northern and western blotting. Total RNA and protein extracted from inoculated leaves at 2 dpi (b) or upper leaves at 2 wk post‐inoculation (wpi) (c) were analyzed by northern and western blotting. Viral RNAs of BrYV were hybridized with a random primed 3′ untranslated region (UTR)‐specific probe. Methylene blue staining of rRNAs after northern transfer was used as a loading control for high‐molecular‐weight RNA blots (rRNA). The bands corresponding to viral genomic RNAs (gRNA) and subgenomic RNAs (sgRNA) are indicated respectively at the right side of the panel. Coat protein of BrYV (CP^B^ ^r^) was detected with BrYV CP polyclonal antiserum (α CP^B^ ^r^). Coomassie stains of total proteins are shown to indicate equal loading (Coom.).

To explore the role that P0^Br^ plays in the BrYV infection process, we generated a BrYV infectious clone with mutations in the P0 protein (Fig. 8a) (Zhang *et al*., 2015). Due to partial overlapping of ORF0 and ORF1 (Fig. 8a), most BrYV representative mutants that we intended to produce, for example BrYV^L184A^ or BrYV^L63A/P64A^, could not be constructed without affecting amino acid sequences of the translation product encoded by ORF1. Therefore, we tested two BrYV mutants, BrYV^Q2A^ and BrYV^FS^, which contain mutations upstream of the ORF1 start codon. The mutant BrYV^Q2A^ contains a mutation in ORF0, which abolishes the functions of P0 protein through changing the Gln‐2 of P0 into Ala, while ORF0 of mutant BrYV^FS^ is prematurely terminated because of a frameshift caused by an insertion mutation (Fig. 8a). In addition, we tested the BrYV^Y61D^ mutant because it encodes a function‐deficient version of P0^Br^ (Y61D) (Fig. S6 and Table 1) caused by a mutation downstream of the ORF1 start codon without any changes in the P1 amino acid sequence (Fig. 8a).

BrYV, BrYV^Q2A^, BrYV^Y61D^, BrYV^FS^ or empty vector were agroinoculated into leaves of 4‐ to 5‐wk‐old *N. benthamiana* (Yao *et al*., 2011). Viral RNAs of wild‐type BrYV could be readily detected in the inoculated leaves at 2 dpi or in the upper leaves at 2 wks post‐inoculation (wpi) (Fig. 8b,c, lane 5). By contrast, although all the BrYV mutants were detectable in the inoculated leaves at 2 dpi, their viral RNA levels were decreased (Fig. 8b, lanes 2–4). Moreover, viral RNAs of BrYV^Q2A^, BrYV^Y61D^ or BrYV^FS^ were undetectable in the upper leaves of inoculated plants at 2 wpi (Fig. 8c, lanes 2–4). Western blot analyses showed that accumulation of the BrYV coat protein (CP) in inoculated leaves or upper leaves was consistent with viral RNA levels (Fig. 8b,c). We then co‐infiltrated the mutant BrYV^FS^ together with P0^Br^ or its mutants into leaves of 4‐ to 5‐wk‐old *N. benthamiana* to determine whether they could increase the accumulation of BrYV^FS^. Viral RNA levels of BrYV^FS^ could reach that of the wild‐type virus in inoculated leaves at 2 dpi when co‐infiltrated with P0^Br^ or its mutants that can suppress local RNA silencing (L184A, LPK or Δ225–249) (Fig. S7). By contrast, P0^Br^ mutants deficient in suppression of local RNA silencing (Q2A, LP, Y61D and Δ224–249) had no effect on the viral RNA level of BrYV^FS^ (Fig. S7). These results demonstrated that P0 protein is required for the efficient systemic infection of BrYV in plants.

## Discussion

The consensus minimal F‐box like motif (LPXX(L/I)) within P0 proteins has long been suggested to be required for RNA silencing suppression, because the mutation of this motif compromises P0's RNA silencing suppression activity (Pazhouhandeh *et al*., 2006). In addition, the F‐box‐like motif of TuYV P0 is required for its interaction with host SKP1 protein, and depletion of SKP1 in *N. benthamiana* promotes resistance to TuYV. Thus, it was proposed that the P0–SKP1 interaction is essential for RNA silencing suppression (Pazhouhandeh *et al*., 2006). However, several studies challenged the requirement of F‐box‐like motif or P0–SKP1 interaction for RNA silencing suppression (Zhuo *et al*., 2014; Almasi *et al*., 2015). A P0 encoded by PLRV‐IM isolate is an VSR that suppresses RNA silencing by targeting AGO1 for degradation. It harbors two F‐box‐like motifs and the second motif is required for its suppression activity, although P0^PL‐IM^ surprisingly fails to interact with NbSKP1 (Zhuo *et al*., 2014), suggesting that suppression activity of P0 is independent of its interaction with SKP1. Furthermore, P0 of cereal yellow dwarf virus RPS (P0^RPS^) suppresses RNA silencing more efficiently than P0 of cereal yellow dwarf virus RPV (P0^RPV^), but P0^PRS^ binds *Arabidopsis* SKP1‐like 2 (ASK2) less efficiently than P0^RPV^ (Almasi *et al*., 2015). We show that the mutant LPK fails to interact with NbSKP1, but triggers the degradation of AGO1 and suppresses local silencing similar to that of wild‐type P0^Br^. Therefore, we propose that the P0–SKP1 interaction is not directly required for P0 to suppress local RNA silencing through AGO1 destabilization, which supports the findings of Zhuo *et al*. (2014) and Almasi *et al*. (2015). However, the possibility remains that the ubiquitination process plays important roles in the AGO1 degradation triggered by P0 or infection of poleroviruses (Derrien *et al*., 2012). The P0^Tu^ and P0^Tu^LP mutant induces accumulation of polyubiquitinated proteins in plants, and an enrichment of polyubiquitin conjugates was observed in AGO1 immunoprecipitates (Csorba *et al*., 2010; Derrien *et al*., 2012). Furthermore, MLN‐4924, a selective inhibitor of the developmentally down‐regulated 8 (NEDD8)/ubiquitin‐related protein 1 (RUB1) conjugation pathway in both mammals and plants (Soucy *et al*., 2009; Hakenjos *et al*., 2011), efficiently inhibited CUL1 neddylation and impaired AGO1 degradation in a P0^Tu^‐expressing *Arabidopsis* transgenic line (Derrien *et al*., 2012), indicating the requirement of ubiquitylation in the AGO1 degradation process. The resistance to TuYV that was provoked through silencing of SKP1 in *N. benthamiana* implied the importance of SCF in polerovirus infection as well (Pazhouhandeh *et al*., 2006). It is possible that P0 served as an F‐box protein and led to the degradation of a virus component or host factor, which is essential for host antiviral resistance, rather than for RNA silencing. Published findings together with the results described here shed light on the mechanism underlying P0‐triggered AGO1 degradation and the role of P0 in polerovirus infection and pathogenicity.

Why does the P0^Br^ mutant LP fail to suppress local RNA silencing if the P0–SKP1 interaction is not essential for the silencing suppression activity? As described above, we noted decreased accumulation of LP protein compared to wild‐type P0^Br^ or other mutants. By expressing a second VSR, inhibitor treatments and VIGS analyses, both the ubiquitin‐proteasome and autophagy pathways were identified as factors responsible for the destabilization of the mutant LP. Based on these results, we hypothesize that the autophagy pathway functions as a double‐edged sword in plant defense systems, as P0 hijacks the plant autophagy pathway to degrade the key factor of RISC, while the autophagy pathway in turn will impair the stability of P0. Numerous studies have focused on the host target of VSRs, but the fate of VSRs themselves has not been explored. Tobacco calmodulin‐like protein, rgs‐CaM, binds to the dsRNA‐binding domains of several VSRs, including TuMV HC‐Pro and CMV 2b, and sequesters them from inhibiting RNA silencing. Consequently, these dsRNA‐binding suppressors with rgs‐CaM appear to be degraded through an autophagy‐like degradation pathway (Nakahara *et al*., 2012). However, P0 binds neither siRNAs nor dsRNAs in *in vitro* assay (Zhang *et al*., 2006; Csorba *et al*., 2010), and the proteasome also contributes to degradation. Further studies are required to identify the host factors that perceive P0 and mediate its degradation.

Many F‐box proteins are targeted for ubiquitin‐dependent degradation through an autocatalytic mechanism. Mutations in the F‐box motifs have been shown to attenuate ubiquitination and degradation of these F‐box proteins (Zhou & Howley, 1998; Galan & Peter, 1999; He *et al*., 2005). By contrast, degradation of several F‐box proteins, including Skp2 of *Homo sapiens*, Dia2 of *Saccharomyces cerevisiae*, COI1 of *Arabidopsis* and SLF1 of *Petunia inflata*, are independent of an autocatalytic mechanism (Kile & Koepp, 2010; Yan *et al*., 2013; Sun *et al*., 2015). Instead of being stable, mutants of such F‐box proteins lacking the F‐box motif were degraded more rapidly, indicating that these F‐box proteins do not need to be part of an SCF complex to be a substrate of degradation. Moreover, ASK1 and integrity of SCF^COI1^ are indicated to be essential for COI1 stability in *Arabidopsis* (Yan *et al*., 2013). Similar to COI1, our findings described here suggest that turnover of BrYV P0 protein is independent of an autocatalytic mechanism. Either mutation of an F‐box‐like motif or knockdown of SKP1 destabilize this viral F‐box‐like protein P0^Br^ and consequently impair its RNA silencing suppression activity with self‐sacrifice, indicating that P0 is targeted either by an unidentified E3 ligase other than its own SCF complex or by an unknown host factor. This implies a possible counterattack strategy developed by BrYV, in which its VSR P0 protein can escape from degradation by means of mimicking a host F‐box protein and interact with SKP1. This raises the question of how SKP1 facilitates stability of P0^Br^. One possibility is that, once P0 forms an SCF^P0^ complex together with SKP1, the recognition sites in P0^Br^ become inaccessible to those interactions that contribute to its degradation, keeping P0^Br^ protected from recognition and subsequent degradation by host factors. This may be attributed to recognition site‐occupying by SKP1, P0 conformational changes or altered post‐translational modification status of P0.

However, we still need to explain the stabilization of mutant LPK that harbors mutations in the F‐box‐like motif. We first inferred that the Lys44 residue serves as a key site of P0^Br^ and its ubiquitination may mediate degradation of P0 through 26S proteasome and autophagy pathways. Nonetheless, when we substituted other lysine residues of P0^Br^ with alanine, all of the mutations rescued the protein stabilization and the local silencing suppression activity of the LP mutant (data not shown). Furthermore, alanine substitution of Leu184 also had the same effect (data not shown). We speculate that alanine substitution of lysine or leucine residues alters the attackable conformation or post‐translational modification status of LP to a stable one with similar features to that of P0^Br^ in the context of P0–SKP1 interaction. Notably, as shown in Fig. S4, although Myc‐tagged BD‐P0, BD‐LP and BD‐LPK were expressed in the Y2H assay, BD‐LP migrates at a lower rate in the gel compared with the others, providing evidence for the possible change in protein conformation or post translational modification status of these mutants in yeast. We have already known that association between P0^Tu^ and AtCUL1 requires SKP1 as a bridging component (Pazhouhandeh *et al*., 2006). It is possible that the alanine substitution of lysine or leucine residues stabilizes LP through establishing a new interaction with one of the other members of the SCF E3 ubiquitin ligase complex, such as direct interaction with CUL1. In addition to facilitating stability of P0, we cannot exclude the possibility that the existence of an F‐box‐like domain in P0 and/or the P0–SKP1 interaction may ensure residues Q2, Y61, Y96 and Y211 become accessible to interaction with AGO1 or other proteins that are directly functionally important for RNA silencing. This assumption may provide an explanation to why the mutation in the F‐box‐like motif did not compromise the protein stabilities of P0 of TuYV or PLRV but impaired their silencing suppression activities (Pazhouhandeh *et al*., 2006; Zhuo *et al*., 2014).

**Figure 9:**
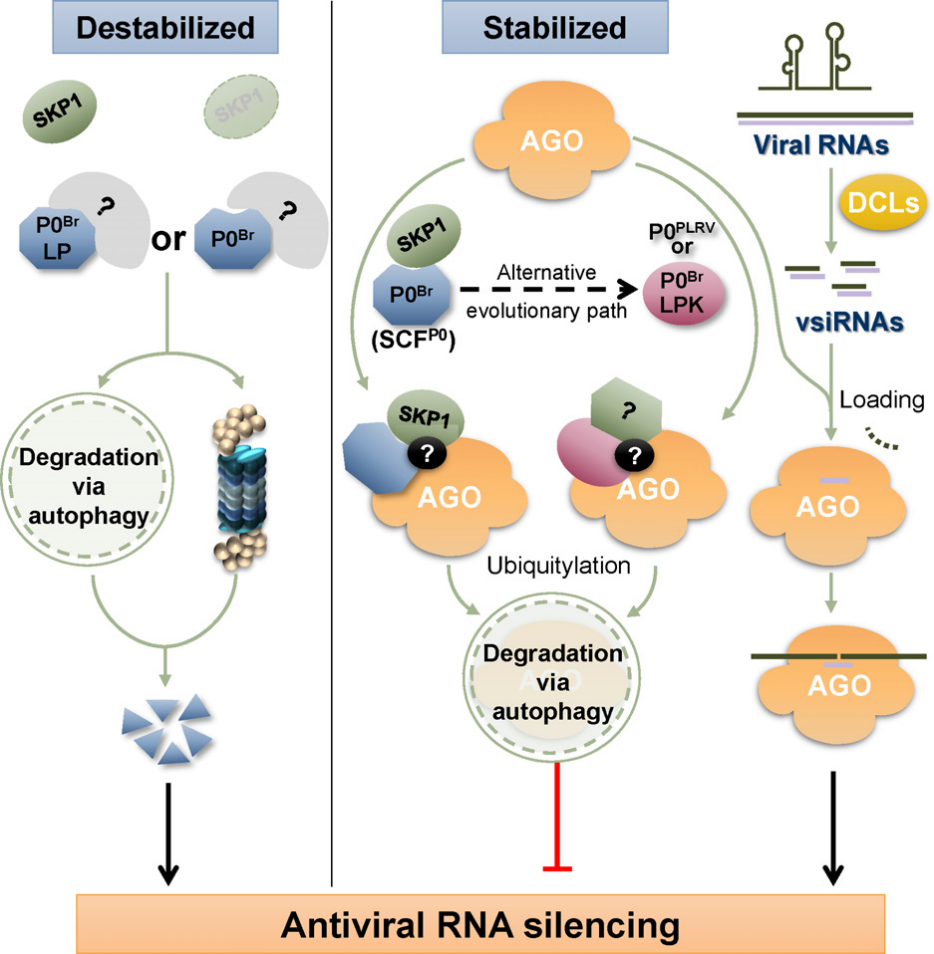
Models for protein stabilization and silencing suppression mechanisms of P0^Br^. P0^Br^ is a viral suppressor of RNA silencing encoded by the polerovirus BrYV. In plant cells, P0^Br^ protein is stabilized when interacting with SKP1 and suppresses local RNA silencing through triggering degradation of AGO1 (the core factor of RISC) by the autophagy pathway. The mutation in the P0^Br^ F‐box‐like motif (LP and LPK) dissociates P0 from the SCF complex. The mutant LP and the dissociated P0^Br^ in *SKP1*‐knockdown plants are likely to be perceived by plant cells and destabilized through both the 26S proteasome system and autophagy pathway, and thus are deficient in local silencing suppression. However, the mutant LPK and the wild‐type P0 encoded by PLRV (P0^PLRV^) are stable and still display silencing suppression activity without interaction with SKP1, indicating that silencing suppression is independent of P0–SKP1 interaction. They may represent an alternative evolutionary trend of polerovirus P0 proteins to maintain self‐stabilization.

In conclusion, we propose a model in which the BrYV VSR protein P0 maintains its own stability and then exerts RNA silencing suppression activity for efficient systemic infection (Fig. 9). In the model, as a pathogenic determinant, the VSR P0^Br^ is likely to be recognized and degraded by host cells through pathways such as ubiquitin–26S proteasome and autophagy. To avoid degradation, P0^Br^ may mimic the host F‐box proteins and interact with SKP1, a key factor in the SCF E3 ligase complex which may further assemble with other factors to form SCF^P0^ complexes. The stabilized SCF^P0^ then associates with and triggers degradation of AGO1 (the core factor of RISC) through the autophagy pathway in a ubiquitylation‐dependent manner to suppress antiviral RNA silencing. Moreover, the mutant LPK and the wild‐type P0 encoded by PLRV (P0^PLRV^) may represent an alternative evolutionary trend of polerovirus P0 to stabilize itself via an unknown strategy, indicating that silencing suppression is independent of P0–SKP1 interaction.

## Author contributions

CH, YL, QS and HX designed the research; YL, QS, HX, ZW, TZ, Xiaoyan Zhang, Xin Zhang and CZ performed the experiments; YL, QS, HX, ZW, TZ, CZ and CH analyzed the data; YL, YW, XW, SPD‐K and CH wrote the paper; DL, JY, XW, YZ and YW contributed through discussions; YL and QS contributed equally to this work.

## Supporting information

Please note: Wiley Blackwell are not responsible for the content or functionality of any Supporting Information supplied by the authors. Any queries (other than missing material) should be directed to the *New Phytologist* Central Office.

**Fig. S1** The Phe219 residue in P0^Br^ is essential for local and systemic silencing suppression.
**Fig. S2** Symptoms of *Nicotiana benthamiana* plants infected with TRV:00 or TRV:*NbATG5*.
**Fig. S3** P0^Br^‐mediated degradation of AGO1 is blocked by E‐64d inhibitor.
**Fig. S4** Detection of Myc‐tagged P0^Br^ and its mutants in yeast. Total proteins were extracted from yeasts.
**Fig. S5** Detection of P0^Br^‐6Myc in *XVE*:*P0* ^*Br*^ *‐6Myc* transgenic *Nicotiana benthamiana* plants.
**Fig. S6** Mutation of the tyrosine‐61 residue in P0^Br^ abolished suppression of RNA silencing.
**Fig. S7** Complementation analysis of P0^Br^ and its mutants.
**Table S1** Sequences of primers and probes.
