# Supplementary material for: Interaction between Brassica yellows virus silencing suppressor P0 and plant SKP1 facilitates stability of P0 in vivo against degradation by proteasome and autophagy pathways
Source: New Phytol. 2019 Feb 26;222(3):1458–73. doi: 10.1111/nph.15702 (PMC6593998; doi:10.1111/nph.15702)
Supplement: Supplementary file 1 — Fig. S1 The Phe219 residue in P0Br is essential for local and systemic silencing suppression. Fig. S2 Symptoms of Nicotiana benthamiana plants infected with TRV:00 or TRV:NbATG5. Fig. S3 P0Br‐mediated degradation of AGO1 is blocked by E‐64d inhibitor. Fig. S4 Detection of Myc‐tagged P0Br and its mutants in yeast. Total proteins were extracted from yeasts. Fig. S5 Detection of P0Br‐6Myc in XVE:P0 Br ‐6Myc transgenic Nicotiana benthamiana plants. Fig. S6 Mutation of the tyrosine‐61 residue in P0Br abolished suppression of RNA silencing. Fig. S7 Complementation analysis of P0Br and its mutants. Table S1 Sequences of primers and probes. [file NPH-222-1458-s001.pdf]

New Phytologist **Supporting Information**

Article title: **Interaction between brassica yellows virus silencing suppressor P0 and plant SKP1 facilitates stability of P0 in vivo against degradation by proteasome and autophagy pathways**

Authors: Yuanyuan Li, Qian Sun, Tianyu Zhao, Haiying Xiang, Xiaoyan Zhang, Zhanyu Wu, Cuiji Zhou, Xin Zhang, Ying Wang, Yongliang Zhang,Xianbing Wang, Dawei Li, Jialin Yu, Savithramma P. Dinesh-Kumar, Chenggui Han

Article acceptance date: 08 January 2019

The following Supporting Information is available for this article:

Fig. S1 The Phe219 residue in $P0^{Br}$ is essential for local and systemic silencing suppression.

Fig. S2 Symptoms of the *Nicotiana benthamiana* plants infected with TRV:00 or TRV:*NbATG5*.

Fig. S3 $P0^{Br}$-mediated degradation of AGO1 is blocked by E-64d inhibitor.

Fig. S4 Detection of Myc-tagged $P0^{Br}$ and its mutants in yeast.

Fig. S5 Detection of $P0^{Br}$-6Myc in *XVE*:$P0^{Br}$*-6Myc* transgenic *Nicotiana benthamiana* plants.

Fig. S6 Mutation of the tyrosine-61 residue in $P0^{Br}$ abolished suppression of RNA silencing.

Fig. S7 Complementation analysis of $P0^{Br}$ and its mutants.

Table S1 Sequences of primers and probes.

Fig. S1 The Phe219 residue in P0^{Br} is essential for local and systemic silencing suppression. (a) Alignment of C-terminus of P0^{Br} from 10 different viruses of *polerovirus*. P0^{Br} and its C-terminus region containing FWR residues are represented by white and black boxes, respectively. FWR residues and Alanine-223 residue are indicated. (b) Suppression of RNA silencing by P0^{Br} mutant F219R. GFP was transiently co-expressed by *Agrobacterium*-infiltration in *Nicotiana benthamiana* or 16c leaves together with empty vector (EV), P0^{Br}-3FLAG or F219R-3FLAG mutants. Fluorescence images of *N. benthamiana* infiltrated leaves were taken under long-wave length UV light at 2 dpi. The number of plants showing systemic silencing were calculated and compared with total number of coinfiltrated plants tested in 3 independent experiments.

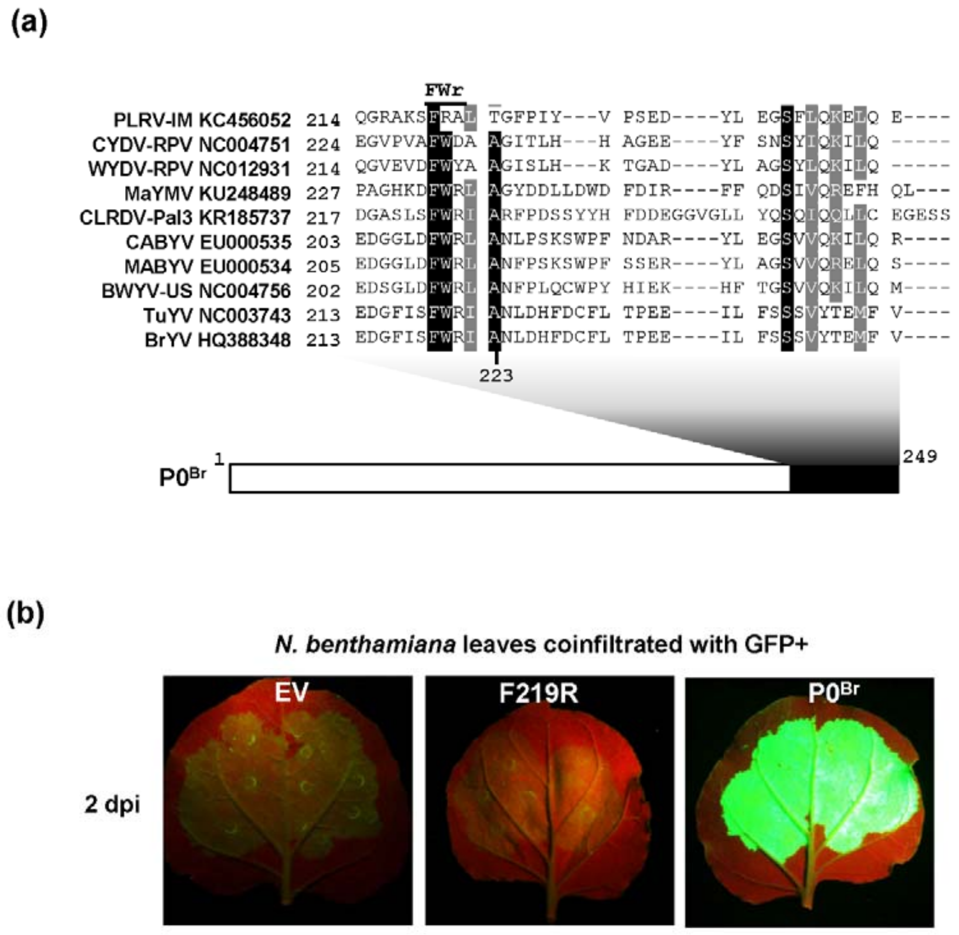

	EV	P0^{Br}	F219R
Silenced/ total	12/12	5/13	12/12
Percentage	100%	38%	100%

Fig. S2 Symptoms of the *Nicotiana benthamiana* plants infected with TRV:00 or TRV:*NbATG5*. Wild-type *N. benthamiana* leaves were inoculated with *Agrobacteria* harboring pTRV1 plus *Agrobacteria* harboring pTRV2 (TRV:00) or pTRV2-*NbATG5* (TRV-*NbATG5*). Photographs of plants were taken 20 dpi.

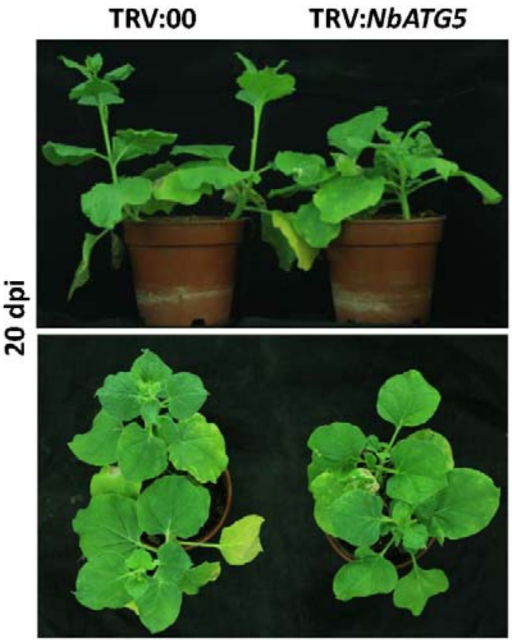

Fig. S3 P0^{Br}-mediated degradation of AGO1 is blocked by E-64d inhibitor. 6Myc-tagged AtAGO1 was transiently co-expressed by *Agrobacterium*-infiltration in *Nicotiana benthamiana* leaves together with 3FLAG-tagged P0^{Br} and its mutant LP in the presence of P38^{TCV}. Total protein was extracted from co-infiltrated patches at 2 dpi. 50 μM E-64d was infiltrated into *N. benthamiana* leaves for 12 h before harvesting (+) and DMSO treatment was used as a solvent control (-). Accumulation of 6Myc-tagged and 3FLAG-tagged proteins were analyzed by western blotting with c-Myc monoclonal antibody (α c-Myc) and FLAG monoclonal antibody (α FLAG), respectively. Coomassie stain of total proteins is shown to indicate equal loading (Coom.).

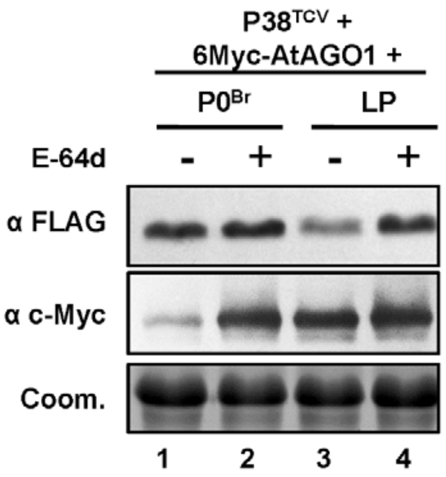

Fig. S4 Detection of Myc-tagged P0^{Br} and its mutants in yeast. Total proteins were extracted from yeasts. Accumulation of myc-tagged proteins were detected by western blotting with c-Myc monoclonal antibody (α c-Myc). Coomassie stains of total proteins are shown to indicate equal loading (Coom.).

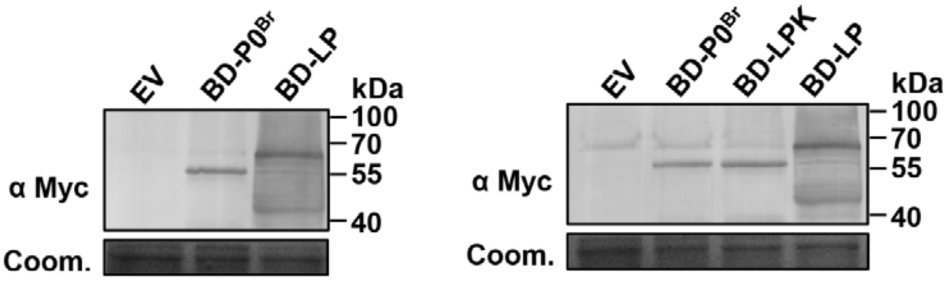

Fig. S5 Detection of $P0^{Br}$-6Myc in *XVE*:*$P0^{Br}$-6Myc* transgenic *Nicotiana benthamiana* plants. Estradiol (100 μM) was applied to the non-transgenic (NT) or *XVE*:*$P0^{Br}$-6Myc* transgenic *N. benthamiana* to induce expression of $P0^{Br}$-6Myc. Leaves were harvested 2 days after estradiol treatment for protein and RNA extraction. Accumulation of 6Myc-tagged P0 proteins were analyzed by western blotting with c-Myc monoclonal antibody (α c-Myc). Coomassie stains of total proteins are shown to indicate equal loading (Coom.).

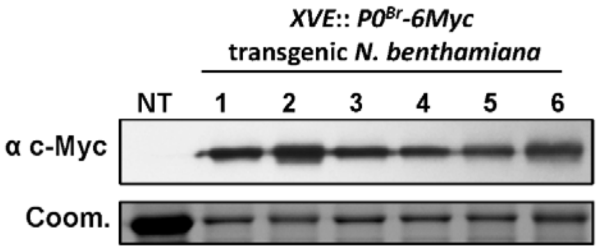

Fig. S6 Mutation of the tyrosine-61 residue in P0^{Br} abolished suppression of RNA silencing. GFP was transiently co-expressed by *Agrobacterium*-infiltration in *Nicotiana benthamiana* leaves together with empty vector (EV), P0^{Br}-3FLAG (P0^{Br}), Y61A-3FLAG, or Y61D-3FLAG. Photographs were taken under long-wave length UV light at 2 dpi.

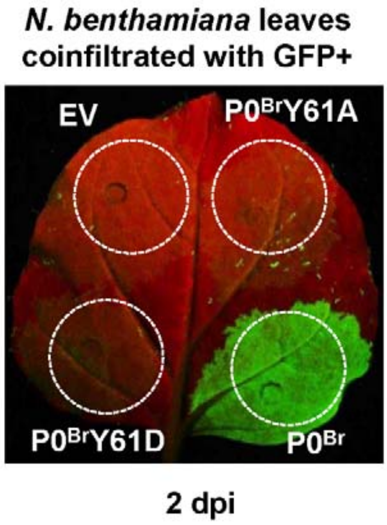

Fig. S7 Complementation analysis of P0^{Br} and its mutants. The mutant BrYV^{FS} was co-infiltreated with 3FLAG-tagged P0^{Br} or its derivative mutants Q2A, LP, L184A, Y61D, Δ224-249, and Δ225-249. Empty vector (EV) co-infiltreated with BrYV^{FS} or BrYV were used as negative and positive control, respectively. Accumulation of the mutant BrYV^{FS} in *Nicotiana benthamiana* plants was analyzed by northern blotting. Total RNA and protein extracted from inoculated leaves at 2 dpi. Viral RNAs of BrYV were hybridized with a random primed 3′ UTR specific probe. Methylene blue staining of ribosomal RNAs after northern transfer was used as loading control for high molecular-weight RNAs blots (rRNA). The bands corresponding to viral genome RNAs (gRNA) and subgenomic RNAs (sgRNA) are indicated respectively at the right side of the panel. 3FLAG-tagged P0^{Br} and its derivative mutants were detected with FLAG monoclonal antibody (α FLAG). Coomassie stain of total proteins is shown to indicate equal loading (Coom.).

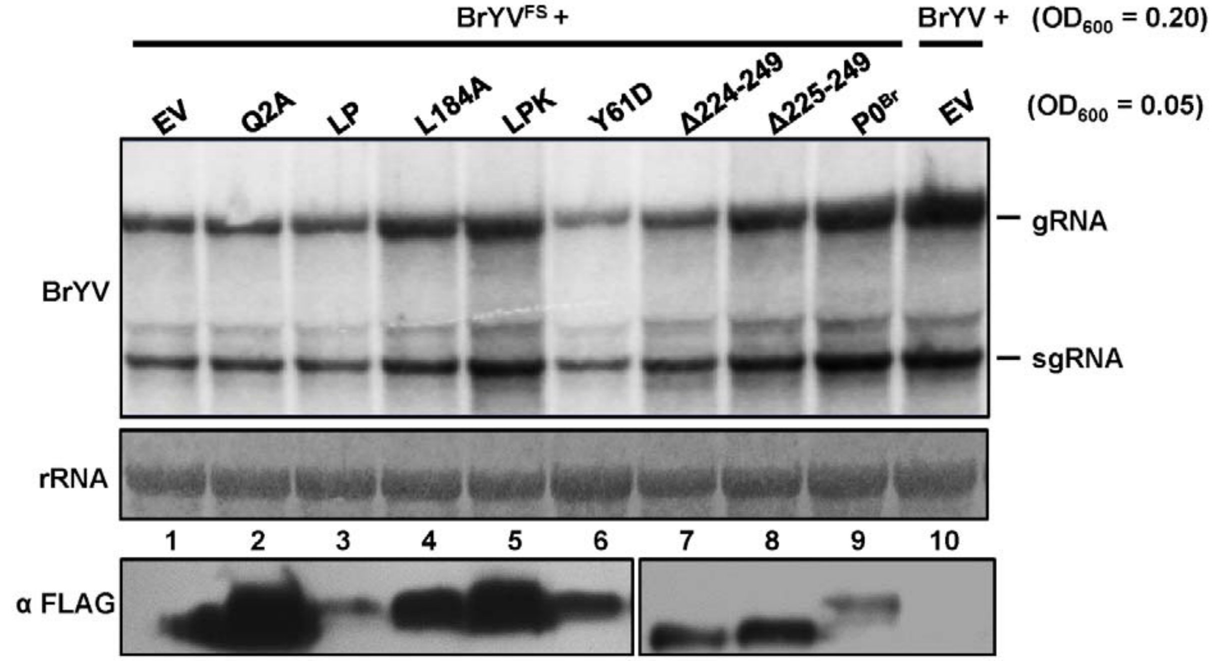

Table S1. Sequences of primers and probes

Experiment	Name	Sequence	Note
**Cloning of expression constructs/ Y2H constructs**	**P38-F**	CCC*aagctt*ATGGAAAATGATCCTAGAG	pGD-P38^{TCV} cloning
	**P38-R**	CGC*ggatcc*CTAAATTCTGAGTGCTTG	
	**BrP0XhoF**	T*ctcgag*ATGCAATTTGTAGC	pGD-P0^{Br}-3FLAG cloning
	**BrP0ApaR**	C*gggccc*TACAAACATTTCGG	
	**BrP0GGGApaR**	AT*gggccc*TCCTCCGCCTACAAACATTTCGG	pGD-P0^{Br}-GFP cloning
	**BrP0Nde1F**	TTC*catatg*ATGCAATTTGTAGCTCAC	pGBK-P0^{Br} and its mutants cloning
	**BrP0BamH1R**	CG*ggatcc*TACAAACATTTCGGTGT	
	**Q2ANde1F**	TTC*catatg*ATGGCATTTGTAGCTCAC	pGBK-P0^{Br}Q2A cloning
	**NbSKP1XhoF**	CCG*ctcgag*ATGAAGATGATCGTGC	pGD-NbSKP1-GFP cloning
	**NbSKP1ApaR**	AC*gggccc*CTCGAAGGCCCAGGC	
	**GUSXhoF**	CG*ctcgag*ATGTTACGTCCTGTAGAAAC	pGD-GUS-3FLAG cloning
	**GUSApaR**	AC*gggccc*TCATTGTTTGCCTCCCTGC	
	**Br-P0XhoF**	*CTCGAG*ATGCAATTCATTGC	pER8-P0^{Br}-6Myc cloning
	**Myc-SpeR**	*ACTAGT*TTACAAGTCTTCTTCTGAG	
**Truncation mutagenesis of P0^{Br}**	**BrP0N2**	TAT*ctcgag*ATGTTTGTAGCTCACG	P0^{Br}Δ2 cloning
	**BrP0N2-3**	TAT*ctcgag*ATGGTAGCTCACGACAAC	P0^{Br}Δ2-3 cloning
	**BrP0N2-4**	TAT*ctcgag*ATGGCTCACGACAACTTTCAC	P0^{Br}Δ2-4 cloning
	**BrP0N3**	TAT*ctcgag*ATGCAAGTAGCTCACG	P0^{Br}Δ3 cloning
	**C223TGA**	CG*ggatccgggccc*TCAGGCAATCCTCCAAAAAG	P0^{Br}Δ224-249 cloning
	**C224TGA**	TAT*ggatccgggccc*TCAATTGGCAATCCT	P0^{Br}Δ225-249 cloning
	**C225TGA**	TAT*gggccc*TCACAGATTGGCAATCCT	P0^{Br}Δ226-249 cloning

Experiment	Name	Sequence	Note
**Alanine substitution mutagenesis of P0^{Br}**	**BrYVAQ2AF(-)**	GTAGCTCACGACAACTTTCAC	P0^{Br}Q2A cloning
	**BrYVAQ2AR(+)**	AAATGCCATAAGCTTATC	
	**BrF3R(-)**	CATAAGCTTATCAAACAAAG	P0^{Br}F3A cloning
	**TuAF3AF(+)**	CAAGCTGTAGCTCACGACAAC	
	**BrD7F(-)**	AACTTTCACACTCTAGAAGTC	P0^{Br}V4A cloning
	**BrV4AR(+)**	GTCGTGAGCTGCAAATTGC	
	**BrD7F(-)**	AACTTTCACACTCTAGAAGTC	P0^{Br}H6A cloning
	**BrH6AR(+)**	GTCTGCAGCTACAAATTGC	
	**BrD7F(-)**	AACTTTCACACTCTAGAAGTC	P0^{Br}D7A cloning
	**BrD7AR(+)**	TGCGTGAGCTACAAATTGC	
	**BrN8AF(+)**	GCATTTCACACTCTAGAAGTC	P0^{Br}N8A cloning
	**BrF9R(-)**	GTCGTGAGCTACAAATTGC	
	**BrF9AF(+)**	AACGCTCACACTCTAGAAGTC	P0^{Br}H10A cloning
	**BrF9R(-)**	GTCGTGAGCTACAAATTGC	
	**BrH10AF(+)**	AACTTTGCAACTCTAGAAGTC	P0^{Br}T11A cloning
	**BrF9R(-)**	GTCGTGAGCTACAAATTGC	
	**BrT11AF(+)**	AACTTTCACGCTCTAGAAGTCAGA	P0^{Br}L12A cloning
	**BrF9R(-)**	GTCGTGAGCTACAAATTGC	
	**BrL12AF(+)**	AACTTTCACACTGCAGAAGTC	P0^{Br}E13A cloning
	**BrF9R(-)**	GTCGTGAGCTACAAATTGC	
	**BrYVAE13AF(+)**	CTAGCAGTCAGAAAAGTTAG	P0^{Br}V14A cloning
	**BrYVA E13AR(-)**	AGTGTGAAAGTTGTCGTG	
	**BrYVAV14AF(+)**	CTAGAAGCAAGAAAAGTTAG	P0^{Br}R15A cloning

Experiment	Name	Sequence	Note
	**BrYVA E13AR(-)**	AGTGTGAAAGTTGTCGTG	
	**BrR15AF(+)**	GCAAAAGTTAGATTCCTCCATC	P0^{Br}R15A cloning
	**BrR15R(-)**	GACTTCTAGAGTGTGAAAGTTG	
	**BrK16AF(+)**	AGAGCAGTTAGATTCCTCCATC	P0^{Br}K16A cloning
	**BrR15R(-)**	GACTTCTAGAGTGTGAAAGTTG	
	**BrV17AF(+)**	AGAAAAGCAAGATTCCTCCATC	P0^{Br}V17A cloning
	**BrR15R(-)**	GACTTCTAGAGTGTGAAAGTTG	
	**BrR18AF(+)**	AGAAAAGTTGCATTCCTCCATC	P0^{Br}R18A cloning
	**BrR15R(-)**	GACTTCTAGAGTGTGAAAGTTG	
	**BrF19AF(+)**	AGAGCCCTCCATCCGCGAC	P0^{Br}F19A cloning
	**BrF19R(-)**	AACTTTTCTGACTTCTAGAG	
	**BrL20AF(+)**	AGAAAAGTTAGATTCGCACATC	P0^{Br}L21A cloning
	**BrR15R(-)**	GACTTCTAGAGTGTGAAAGTTG	
	**BrH21AF(+)**	AGAAAAGTTAGATTCCTCGCACC	P0^{Br}H21A cloning
	**BrR15R(-)**	GACTTCTAGAGTGTGAAAGTTG	
	**BrV25F(-)**	ACGTTTCTTTTAGCAGGTTTA	P0^{Br}R23A cloning
	**BrR23AR(+)**	TACTTGTGCCGGATGGAGG	
	**BrV25F(-)**	ACGTTTCTTTTAGCAGGTTTA	P0^{Br}Q24A cloning
	**BrQ24AR(+)**	TACTGCTCGCGGATGGAGG	
	**BrV25F(-)**	ACGTTTCTTTTAGCAGGTTTA	P0^{Br}V25A cloning
	**BrV25AR(+)**	TGCTTGTCGCGGATGGAGG	
	**BrT26AF(+)**	GCGTTTCTTTTAGCAGGTT	P0^{Br}T26A cloning
	**BrF27R(-)**	TACTTGTCGCGGATGGAGG	

Experiment	Name	Sequence	Note
	**BrF27AF(+)**	ACGGCTCTTTTAGCAGGTTTAT	P0^Br^F27A cloning
	**BrF27R(-)**	TACTTGTCGCGGATGGAGG	
	**BrL28AF(+)**	ACGTTTGCATTAGCAGGTTTA	P0^Br^L28A cloning
	**BrF27R(-)**	TACTTGTCGCGGATGGAGG	
	**BrL29AF(+)**	ACGTTTCTTGCAGCAGGTTTA	P0^Br^L29A cloning
	**BrF27R(-)**	TACTTGTCGCGGATGGAGG	
	**BrG31AF(+)**	ACGTTTCTTTTAGCAGCATTA	P0^Br^G31A cloning
	**BrF27R(-)**	TACTTGTCGCGGATGGAGG	
	**BrYVAL32AF(+)**	GGTGCATTGCTTAACATC	P0^Br^L32A cloning
	**BrYVAL32AR(-)**	TGCTAAAAGAAACGTTAC	
	**BrYVAL33AF(+)**	GGTTTAGCACTTAACATC	P0^Br^L33A cloning
	**BrYVAL32AR(-)**	TGCTAAAAGAAACGTTAC	
	**BrYVAL34AF(+)**	GGTTTATTGGCAAACATC	P0^Br^L34A cloning
	**BrYVAL32AR(-)**	TGCTAAAAGAAACGTTAC	
	**BrK41F(-)**	AAAGCAATCAAAGAGCGCAAC	P0^Br^N35A cloning
	**BrN35AR(+)**	TACGAATTGTTCGATTGCAAGCAA	
	**BrK41F(-)**	AAAGCAATCAAAGAGCGCAAC	P0^Br^I36A cloning
	**BrI36AR(+)**	TACGAATTGTTCTGCGTTAAGCAA	
	**BrK41F(-)**	AAAGCAATCAAAGAGCGCAAC	P0^Br^E37A cloning
	**BrE37AR(+)**	TACGAATTGTGCGATGTTAAGCAA	
	**BrK41F(-)**	AAAGCAATCAAAGAGCGCAAC	P0^Br^Q38A cloning
	**BrQ38AR(+)**	TACGAATGCTTCGATGTTAAGCAA	
	**BrF39AF(+)**	CAAGCCGTAAAAGCAATCAAAG	P0^Br^F39A cloning

Experiment	Name	Sequence	Note
	**BrF39R(-)**	TTCGATGTTAAGCAATAAAC	
	**BrK41F(-)**	AAAGCAATCAAAGAGCGCAAC	$P0^{Br}$V40A cloning
	**BrV40AR(+)**	TGCGAATTGTTCGATGTTAAGCAA	
	**BrK41AF(+)**	GCAGCAATCAAAGAGCGCAAC	$P0^{Br}$K41A cloning
	**BrK41R(-)**	TACGAATTGTTCGATGTTAAGCAA	
	**BrI43AF(+)**	AAAGCAGCAAAAGAGCGCAAC	$P0^{Br}$I43A cloning
	**BrK41R(-)**	TACGAATTGTTCGATGTTAAGCAA	
	**BrK44AF(+)**	AAAGCAATCGCAGAGCGCAAC	$P0^{Br}$K44A and $P0^{Br}$LPK cloning
	**BrK41R(-)**	TACGAATTGTTCGATGTTAAGCAA	
	**BrE45AF(+)**	AAAGCAATCAAAGCACGCAAC	$P0^{Br}$E45A cloning
	**BrK41R(-)**	TACGAATTGTTCGATGTTAAGCAA	
	**BrYVAR46AF(+)**	AAGAGGCAAACAATGAATTC	$P0^{Br}$R46A cloning
	**BrYVAR46AR(-)**	TGATTGCTTTTACGAATTGTTC	
	**BrYVAN47AF(+)**	AAGAGCGCGCAAATGAATTC	$P0^{Br}$N47A cloning
	**BrYVAR46AR(-)**	TGATTGCTTTTACGAATTGTTC	
	**BrN48AF(+)**	GCCGAATTCAAGATTGATATTTTTATTCGC	$P0^{Br}$N48A cloning
	**BrN48R(-)**	GTTGCGCTCTTTGATTGCTTTTACG	
	**BrE49AF(+)**	AATGCCTTCAAGATTGATATTTTTATTCGC	$P0^{Br}$E49A cloning
	**BrN48R(-)**	GTTGCGCTCTTTGATTGCTTTTACG	
	**BrF50AF(+)**	GAAGCCAAGATTGATATTTTTA	$P0^{Br}$F50A cloning
	**BrF50R(-)**	ATTGTTGCGCTCTTTGATTGC	
	**BrK51AF(+)**	AATGAATTCGCCATTGATATTTTTATTCGC	$P0^{Br}$K51A cloning
	**BrN48R(-)**	GTTGCGCTCTTTGATTGCTTTTACG	

Experiment	Name	Sequence	Note
	**BrI52AF(+)**	AATGAATTCAAGGCTGATATTTTTATTCGC	P0^{Br}I52A cloning
	**BrN48R(-)**	GTTGCGCTCTTTGATTGCTTTTACG	
	**BrD53AF(+)**	AATGAATTCAAGATTGCCATTTTTATTCGC	P0^{Br}D53A cloning
	**BrN48R(-)**	GTTGCGCTCTTTGATTGCTTTTACG	
	**BrI54AF(+)**	AATGAATTCAAGATTGATGCTTTTATTCGC	P0^{Br}I54A cloning
	**BrN48R(-)**	GTTGCGCTCTTTGATTGCTTTTACG	
	**BrF55AF(+)**	ATTGCTATTCGCTCTCTGC	P0^{Br}F55A cloning
	**BrF55R(-)**	ATCAATCTTGAATTCATTGTTG	
	**BrI56AF(+)**	AATGAATTCAAGATTGATATTTTTGCTCGC	P0^{Br}I56A cloning
	**BrN48R(-)**	GTTGCGCTCTTTGATTGCTTTTACG	
	**BrR57AF(+)**	GCATCTCTGCTCTATCAGCTTCCTCTCCTTC	P0^{Br}R57A cloning
	**BrS58R(-)**	AATAAAAATATCAATCTTGAAT	
	**BrS58AF(+)**	CGCGCTCTGCTCTATCAGCTTC	P0^{Br}S58A cloning
	**BrS58R(-)**	AATAAAAATATCAATCTTGAAT	
	**BrL59AF(+)**	CGCTCTGCACTCTATCAGCTTCCTCTCCTTC	P0^{Br}L59A cloning
	**BrS58R(-)**	AATAAAAATATCAATCTTGAAT	
	**BrL60AF(+)**	CGCTCTCTGGCATATCAGCTTCCTCTCCTTC	P0^{Br}L60A cloning
	**BrS58R(-)**	AATAAAAATATCAATCTTGAAT	
	**BrY61AF(+)**	CGCTCTCTGCTCGCTCAGCTTCCTCTCC	P0^{Br}Y61A cloning
	**BrS58R(-)**	AATAAAAATATCAATCTTGAAT	
	**BrYVAQ62AF(+)**	GCACTTCCTCTCCTTCTCGG	P0^{Br}Q62A cloning
	**BrYVAQ62AR(-)**	ATAGAGCAGAGAGCGAAT	
	**BrL63AF(+)**	CGCTCTCTGCTCTATCAGGCACCTCTCCTTC	P0^{Br}L63A cloning

Experiment	Name	Sequence	Note
	**BrS58R(-)**	AATAAAAATATCAATCTTGAAT	
	**BrYVAL65AF(+)**	CAGCTTCCTGCACTTCTCGG	$\mathrm{P0^{Br}}$L65A cloning
	**BrYVAQ62AR(-)**	ATAGAGCAGAGAGCGAAT	
	**BrYVAL66AF(+)**	CAGCTTCCTCTCGCACTCGG	$\mathrm{P0^{Br}}$L66A cloning
	**BrYVAQ62AR(-)**	ATAGAGCAGAGAGCGAAT	
	**BrL67AF(+)**	GCAGGAGACCACGTCCACGATGACGTTAGG	$\mathrm{P0^{Br}}$L67A cloning
	**BrL67R(-)**	AAGGAGAGGAAGCTGATAGAGCAG	
	**BrG68AF(+)**	CTCGCAGACCACGTCCACGATGACGTTAGG	$\mathrm{P0^{Br}}$G68A cloning
	**BrL67R(-)**	AAGGAGAGGAAGCTGATAGAGCAG	
	**BrD69AF(+)**	CTCGGAGCACACGTCCACGATGACGTTAGG	$\mathrm{P0^{Br}}$D69A cloning
	**BrL67R(-)**	AAGGAGAGGAAGCTGATAGAGCAG	
	**BrH70AF(+)**	CTCGGAGACGCAGTCCACGATGACGTTAGG	$\mathrm{P0^{Br}}$H70A cloning
	**BrL67R(-)**	AAGGAGAGGAAGCTGATAGAGCAG	
	**BrV71AF(+)**	CTCGGAGACCACGCACACGATGACGTTAGG	$\mathrm{P0^{Br}}$V71A cloning
	**BrL67R(-)**	AAGGAGAGGAAGCTGATAGAGCAG	
	**BrH72AF(+)**	CTCGGAGACCACGTCGCAGATGACGTTAGG	$\mathrm{P0^{Br}}$H72A cloning
	**BrL67R(-)**	AAGGAGAGGAAGCTGATAGAGCAG	
	**BrD73AF(+)**	CTCGGAGACCACGTCCACGCAGACGTTAGG	$\mathrm{P0^{Br}}$D73A cloning
	**BrL67R(-)**	AAGGAGAGGAAGCTGATAGAGCAG	
	**BrD74AF(+)**	CTCGGAGACCACGTCCACGATGCAGTTAGG	$\mathrm{P0^{Br}}$D74A cloning
	**BrL67R(-)**	AAGGAGAGGAAGCTGATAGAGCAG	
	**BrV75AF(+)**	CTCGGAGACCACGTCCACGATGACGCAAGG	$\mathrm{P0^{Br}}$V75A cloning
	**BrL67R(-)**	AAGGAGAGGAAGCTGATAGAGCAG	

Experiment	Name	Sequence	Note
	**BrYVAR76AF(+)**	CGTTGCAAAGTCCATACTTGTC	P0^{Br}R76A cloning
	**BrYVAR76AR(-)**	TCATCGTGGACGTGGTCTCC	
	**BrK77AF(+)**	GCATCCATACTTGTCCCTGAACCA	P0^{Br}K77A cloning
	**BrS78R(-)**	CCTAACGTCATCGTGGACGTG	
	**BrS78AF(+)**	AAGGCCATACTTGTCCCTGAAC	P0^{Br}S78A cloning
	**BrS78R(-)**	CCTAACGTCATCGTGGACGTG	
	**BrI79AF(+)**	AAGTCCGCACTTGTCCCTGAACCA	P0^{Br}I79A cloning
	**BrS78R(-)**	CCTAACGTCATCGTGGACGTG	
	**BrL80AF(+)**	AAGTCCATAGCAGTCCCTGAACCA	P0^{Br}L80A cloning
	**BrS78R(-)**	CCTAACGTCATCGTGGACGTG	
	**BrV81AF(+)**	AAGTCCATACTTGCCCCTGAACCA	P0^{Br}V81A cloning
	**BrS78R(-)**	CCTAACGTCATCGTGGACGTG	
	**BrE83AF(+)**	AAGTCCATACTTGTCCCTGCACCA	P0^{Br}E83A cloning
	**BrS78R(-)**	CCTAACGTCATCGTGGACGTG	
	**BrE85AF(+)**	GCACTCTGCGCCTGGTTCTCTTTAC	P0^{Br}E85A cloning
	**BrE85R(-)**	TGGTTCAGGGACAAGTATGGAC	
	**BrL86AF(+)**	GAGGCAGCGCCTGGTTCTCTTTAC	P0^{Br}L86A cloning
	**BrE85R(-)**	TGGTTCAGGGACAAGTATGGAC	
	**BrC87AF(+)**	GAGCTCGCAGCCTGGTTCTCTTTAC	P0^{Br}C87A cloning
	**BrE85R(-)**	TGGTTCAGGGACAAGTATGGAC	
	**BrW89AF(+)**	GAGCTCTGCGCCGCATTCTCTTTAC	P0^{Br}W89A cloning
	**BrE85R(-)**	TGGTTCAGGGACAAGTATGGAC	
	**BrF90AF(+)**	TGGGCCTCTTTACAAACGGG	P0^{Br}F90A cloning

Experiment	Name	Sequence	Note
	**BrF90R(-)**	GGCGCAGAGCTCTGGTTCAG	
	**BrS91AF(+)**	GCTTTACAAACGGGATATGC	P0^Br^S91A cloning
	**BrS91R(-)**	GAACCAGGCGCAGAGCTCTGG	
	**BrYVAL92AF(+)**	TCTGCACAAACGGGATATG	P0^Br^L92A cloning
	**BrYVAL92AR(-)**	GAACCAGGCGCAGAGCTCTG	
	**BrQ93AF(+)**	TCTTTAGCAACGGGATATGCTCCC	P0^Br^Q93A cloning
	**BrS91R(-)**	GAACCAGGCGCAGAGCTCTGG	
	**BrT94AF(+)**	TCTTTACAAGCGGGATATGCTCCCG	P0^Br^T94A cloning
	**BrS91R(-)**	GAACCAGGCGCAGAGCTCTGG	
	**BrG95AF(+)**	TCTTTACAAACGGCATATGCTCCC	P0^Br^G95A cloning
	**BrS91R(-)**	GAACCAGGCGCAGAGCTCTGG	
	**BrY96AF(+)**	TCTTTACAAGCGGGAGCTGCTCCCGCCTCC	P0^Br^Y96A cloning
	**BrS91R(-)**	GAACCAGGCGCAGAGCTCTGG	
	**BrS100AF(+)**	GCCGCCACCTCAGGCCGTG	P0^Br^S100A cloning
	**BrS100R(-)**	GGGAGCATATCCCGTTTGTA	
	**BrT101AF(+)**	GCCTCCGCCTCAGGCCGTGTTA	P0^Br^T101A cloning
	**BrS100R(-)**	GGGAGCATATCCCGTTTGTA	
	**BrS102AF(+)**	GCCTCCACCGCAGGCCGTGTTAACT	P0^Br^S102A cloning
	**BrS100R(-)**	GGGAGCATATCCCGTTTGTA	
	**BrG103AF(+)**	GCCTCCACCTCAGCACGTGTTAAC	P0^Br^G103A cloning
	**BrS100R(-)**	GGGAGCATATCCCGTTTGTA	
	**BrR104AF(+)**	GCCTCCACCTCAGGCGCAGTTAAC	P0^Br^R104A cloning
	**BrS100R(-)**	GGGAGCATATCCCGTTTGTA	

Experiment	Name	Sequence	Note
	**BrV105AF(+)**	GCCTCCACCTCAGGCCGTGCAAAC	P0^Br^V105A cloning
	**BrS100R(-)**	GGGAGCATATCCCGTTTGTA	
	**BrYVAN106AF(+)**	TGCATTACACGTGCCAGG	P0^Br^N106A cloning
	**BrYVAN106AR(-)**	ACACGGCCTGAGGTGGAGG	
	**BrYVAL107AF(+)**	TAACGCACACGTGCCAGG	P0^Br^L107A cloning
	**BrYVAN106AR(-)**	ACACGGCCTGAGGTGGAGG	
	**BrYVAH108AF(+)**	TAACTTAGCAGTGCCAGG	P0^Br^H108A cloning
	**BrYVAN106AR(-)**	ACACGGCCTGAGGTGGAGG	
	**BrK113F(-)**	ACCTCTCGTAGAAGAATC	P0^Br^V109A cloning
	**BrV109AR(+)**	CTTGGTTCCTGGCGCGTGTAA	
	**BrK113F(-)**	ACCTCTCGTAGAAGAATC	P0^Br^G111A cloning
	**BrG111AR(+)**	CTTGGTTGCTGGCACGTGTAA	
	**BrT112AF(+)**	GGAGCCAAGACCTCTCGTAG	P0^Br^T112A cloning
	**BrT112R(-)**	TGGCACGTGTAAGTTAAC	
	**BrK113F(-)**	ACCTCTCGTAGAAGAATC	P0^Br^K113A cloning
	**BrK113AR(+)**	TGCGGTTCCTGGCACGTGTAA	
	**BrS115AF(+)**	ACCGCTCGTAGAAGAATCAT	P0^Br^S115A cloning
	**BrS115R(-)**	CTTGGTTCCTGGCACGTGTA	
	**BrR116AF(+)**	ACCTCTGCAAGAAGAATCATACAAC	P0^Br^R116A cloning
	**BrS115R(-)**	CTTGGTTCCTGGCACGTGTA	
	**BrR117AF(+)**	ACCTCTCGTGCAAGAATCATACAAC	P0^Br^R117A cloning
	**BrS115R(-)**	CTTGGTTCCTGGCACGTGTA	
	**BrR118AF(+)**	ACCTCTCGTAGAGCAATCATACAAC	P0^Br^R118A cloning

Experiment	Name	Sequence	Note
	**BrS115R(-)**	CTTGGTTCCTGGCACGTGTA	
	**BrI119AF(+)**	ACCTCTCGTAGAAGAGCCATACAAC	P0^{Br}I119A cloning
	**BrS115R(-)**	CTTGGTTCCTGGCACGTGTA	
	**BrI120AF(+)**	ACCTCTCGTAGAAGAATCGCACAAC	P0^{Br}I120A cloning
	**BrS115R(-)**	CTTGGTTCCTGGCACGTGTA	
	**BrYVAQ121AF(+)**	GCACGATCTCTTGCGAGC	P0^{Br}Q121A cloning
	**BrYVAQ121AR(-)**	TATGATTCTTCTACGAGAG	
	**BrR122AF(+)**	GCATCTCTTGCGAGCAATTTC	P0^{Br}R122A cloning
	**BrS123R(-)**	TTGTATGATTCTTCTACGAGAG	
	**BrS123AF(+)**	CGAGCTCTTGCGAGCAATTTC	P0^{Br}S123A cloning
	**BrS123R(-)**	TTGTATGATTCTTCTACGAGAG	
	**BrL124AF(+)**	CGATCTGCAGCGAGCAATTTC	P0^{Br}L124A cloning
	**BrS123R(-)**	TTGTATGATTCTTCTACGAGAG	
	**BrS126AF(+)**	CGATCTCTTGCGGCCAATTTCTCAGAAAAG	P0^{Br}S126A cloning
	**BrS123R(-)**	TTGTATGATTCTTCTACGAGAG	
	**BrYVAN127AF(+)**	AGCGCATTCTCAGAAAAG	P0^{Br}N127A cloning
	**BrYVA N127AR(-)**	CGCAAGAGATCGTTGTATG	
	**BrF128AF(+)**	AATGCCTCAGAAAAGTTCAAG	P0^{Br}F128A cloning
	**BrF128R(-)**	GCTCGCAAGAGATCGTTGTATG	
	**BrS129AF(+)**	CGATCTCTTGCGAGCAATTTCGCAGAAAAGTTCAAG	P0^{Br}S129A cloning
	**BrS123R(-)**	TTGTATGATTCTTCTACGAGAG	
	**BrE130AF(+)**	GCAAAGTTCAAGCGATTTCCAGAATGTTTA	P0^{Br}E130A cloning
	**BrE130R(-)**	TGAGAAATTGCTCGCAAGAGATCG	

Experiment	Name	Sequence	Note
	**BrK131AF(+)**	GAAGCATTCAAGCGATTTCCAGAATGTTTA	P0^{Br}K131A cloning
	**BrE130R(-)**	TGAGAAATTGCTCGCAAGAGATCG	
	**BrF132AF(+)**	AAGGCCAAGCGATTTCCAGAA	P0^{Br}F132A cloning
	**BrF132R(-)**	TTCTGAGAAATTGCTCGCAAG	
	**BrK133AF(+)**	GAAAAGTTCGCACGATTTCCAGAATGTTTA	P0^{Br}K133A cloning
	**BrE130R(-)**	TGAGAAATTGCTCGCAAGAGATCG	
	**BrR134AF(+)**	GAAAAGTTCAAGGCATTTCCAGAATGTTTA	P0^{Br}R134A cloning
	**BrE130R(-)**	TGAGAAATTGCTCGCAAGAGATCG	
	**BrF135AF(+)**	CGAGCTCCAGAATGTTTATTC	P0^{Br}F135A cloning
	**BrF135R(-)**	CTTGAACTTTTCTGAGAAATTG	
	**BrE137AF(+)**	GAAAAGTTCAAGCGATTTCCAGCATGTTTA	P0^{Br}E137A cloning
	**BrE130R(-)**	TGAGAAATTGCTCGCAAGAGATCG	
	**BrYVAC138AF(+)**	GCATTATTCGTTGGCCTTG	P0^{Br}C138A cloning
	**BrYVAC138AR(-)**	TTCTGGAAATCGCTTGAAC	
	**BrYVAL139AF(+)**	TGTGCATTCGTTGGCCTTG	P0^{Br}L139A cloning
	**BrYVAC138AR(-)**	TTCTGGAAATCGCTTGAAC	
	**BrF140AF(+)**	TTAGCCGTTGGCCTTGAACA	P0^{Br}F140A cloning
	**BrF140R(-)**	ACATTCTGGAAATCGCTTGAAC	
	**BrV141AF(+)**	GCAGGCCTTGAACATTTCCAGCGA	P0^{Br}V141A cloning
	**BrV141R(-)**	GAATAAACATTCTGGAAATCG	
	**BrG142AF(+)**	GTTGCACTTGAACATTTCCAGCGA	P0^{Br}G142A cloning
	**BrV141R(-)**	GAATAAACATTCTGGAAATCG	
	**BrL143AF(+)**	GTTGGCGCAGAACATTTCCAGCGA	P0^{Br}L143A cloning

Experiment	Name	Sequence	Note
	**BrV141R(-)**	GAATAAACATTCTGGAAATCG	
	**BrE144AF(+)**	GTTGGCCTTGCACATTTCCAGCGA	P0^Br^E144A cloning
	**BrV141R(-)**	GAATAAACATTCTGGAAATCG	
	**BrH145AF(+)**	GTTGGCCTTGAAGCATTCCAGCGA	P0^Br^H145A cloning
	**BrV141R(-)**	GAATAAACATTCTGGAAATCG	
	**BrF146AF(+)**	GCCCAGCGATTTCTGTCTAC	P0^Br^F146A cloning
	**BrF146R(-)**	ATGTTCAAGGCCAACGAATAAAC	
	**BrYVAQ147AF(+)**	GCACGATTTCTGTCTACTTG	P0^Br^Q147A cloning
	**BrYVAQ147AR(-)**	GAAATGTTCAAGGCCAAC	
	**BrYVAR148AF(+)**	CAGGCATTTCTGTCTACTTG	P0^Br^R148A cloning
	**BrYVAQ147AR(-)**	GAAATGTTCAAGGCCAAC	
	**BrF149AF(+)**	CGAGCTCTGTCTACTTGGAC	P0^Br^F149A cloning
	**BrF149R(-)**	CTGGAAATGTTCAAGGCCAAC	
	**BrL150AF(+)**	GCATCTACTTGGACTAGAGATGC	P0^Br^L150A cloning
	**BrS151R(-)**	AAATCGCTGGAAATGTTCAAG	
	**BrS151AF(+)**	CTGGCCACTTGGACTAGAGA	P0^Br^S151A cloning
	**BrS151R(-)**	AAATCGCTGGAAATGTTCAAG	
	**BrT152AF(+)**	CTGTCTGCTTGGACTAGAGATG	P0^Br^T152A cloning
	**BrS151R(-)**	AAATCGCTGGAAATGTTCAAG	
	**BrW153AF(+)**	CTGTCTACTGCAACTAGAGATGC	P0^Br^W153A cloning
	**BrS151R(-)**	AAATCGCTGGAAATGTTCAAG	
	**BrT154AF(+)**	CTGTCTACTTGGGCTAGAGATGCTGAAAGAC	P0^Br^T154A cloning
	**BrS151R(-)**	AAATCGCTGGAAATGTTCAAG	

Experiment	Name	Sequence	Note
	**BrR155AF(+)**	CTGTCTACTTGGACTGCAGATGC	P0^{Br}R155A cloning
	**BrS151R(-)**	AAATCGCTGGAAATGTTCAAG	
	**BrD156AF(+)**	CTGTCTACTTGGACTAGAGCAGC	P0^{Br}D156A cloning
	**BrS151R(-)**	AAATCGCTGGAAATGTTCAAG	
	**BrR160AF(+)**	GAAAGAGCACTCTTTCCTGGCTG	P0^{Br}E158A cloning
	**BrE158R(-)**	AGCATCTCTAGTCCAAGTAG	
	**BrL161AF(+)**	GAAAGACGCGCATTTCCTGGCTG	P0^{Br}L161A cloning
	**BrE158R(-)**	AGCATCTCTAGTCCAAGTAG	
	**BrF162AF(+)**	CTCGCTCCTGGCTGTCGAGAAA	P0^{Br}F162A cloning
	**BrF162R(-)**	GCGTCTTTCAGCATCTCTAG	
	**BrYVAG164AF(+)**	CCTGCATGTCGAGAAATTCC	P0^{Br}G164A cloning
	**BrYVAG164AR(-)**	AAAGAGGCGTCTTTCAGC	
	**BrC165AF(+)**	GAAAGACGCCTCTTTCCTGGCGCTCGAG	P0^{Br}C165A cloning
	**BrE158R(-)**	AGCATCTCTAGTCCAAGTAG	
	**BrYVAR166AF(+)**	CCTGGCTGTGCAGAAATTCC	P0^{Br}R166A cloning
	**BrYVAG164AR(-)**	AAAGAGGCGTCTTTCAGC	
	**BrE167AF(+)**	GCAATTCCTGTGGGGTCTCACACG	P0^{Br}E167A cloning
	**BrE167R(-)**	TCGACAGCCAGGAAAGAGGCG	
	**BrI168AF(+)**	GAAGCACCTGTGGGGTCTCACACG	P0^{Br}I168A cloning
	**BrE167R(-)**	TCGACAGCCAGGAAAGAGGCG	
	**BrV170AF(+)**	GAAATTCCTGCAGGGTCTCACACG	P0^{Br}V170A cloning
	**BrE167R(-)**	TCGACAGCCAGGAAAGAGGCG	
	**BrG171AF(+)**	GAAATTCCTGTGGCATCTCACACG	P0^{Br}G171A cloning

Experiment	Name	Sequence	Note
	**BrE167R(-)**	TCGACAGCCAGGAAAGAGGCG	
	**BrS172AF(+)**	GGGGCTCACACGCTTGTGGA	P0^Br^S172A cloning
	**BrS172R(-)**	CACAGGAATTTCTCGACAGC	
	**BrH173AF(+)**	GAAATTCCTGTGGGGTCTGCAACG	P0^Br^H173A cloning
	**BrE167R(-)**	TCGACAGCCAGGAAAGAGGCG	
	**BrT174AF(+)**	GGGTCTCACGCGCTTGTGGAGCTCGC	P0^Br^T174A cloning
	**BrS172R(-)**	CACAGGAATTTCTCGACAGC	
	**BrL175AF(+)**	GCAGTGGAGCTCGCTAATGTTGGC	P0^Br^L175A cloning
	**BrL175R(-)**	CGTGTGAGACCCCACAGGAATTTCTCGAC	
	**BrV176AF(+)**	CTTGCAGAGCTCGCTAATGTTGGC	P0^Br^V176A cloning
	**BrL175R(-)**	CGTGTGAGACCCCACAGGAATTTCTCGAC	
	**BrE177AF(+)**	CTTGTGGCACTCGCTAATGTTGGC	P0^Br^E177 cloning
	**BrL175R(-)**	CGTGTGAGACCCCACAGGAATTTCTCGAC	
	**BrL178AF(+)**	CTTGTGGAGGCAGCTAATGTTGGC	P0^Br^L178A cloning
	**BrL175R(-)**	CGTGTGAGACCCCACAGGAATTTCTCGAC	
	**BrN180AF(+)**	CTTGTGGAGCTCGCTGCAGTTGGC	P0^Br^N180A cloning
	**BrL175R(-)**	CGTGTGAGACCCCACAGGAATTTCTCGAC	
	**BrYVAV181AF(+)**	GCAGGCGAGCTTCTCCGC	P0^Br^V181A cloning
	**BrYVAV181AR(-)**	ATTAGCGAGCTCCACAAGC	
	**BrYVAG182AF(+)**	GTTGCAGAGCTTCTCCGC	P0^Br^G182A cloning
	**BrYVAV181AR(-)**	ATTAGCGAGCTCCACAAGC	
	**BrE183AF(+)**	GCACTTCTCCGCGTTATGGTGGC	P0^Br^E183A cloning
	**BrE183R(-)**	GCCAACATTAGCGAGCTCCACAAG	

Experiment	Name	Sequence	Note
	**BrL184AF(+)**	GAGGCACTCCGCGTTATGGTGGC	P0BrL184A cloning
	**BrE183R(-)**	GCCAACATTAGCGAGCTCCACAAG	
	**BrL185AF(+)**	GAGCTTGCACGCGTTATGGTGGC	P0BrL185A cloning
	**BrE183R(-)**	GCCAACATTAGCGAGCTCCACAAG	
	**BrR186AF(+)**	GAGCTTCTCGCAGTTATGGTGGC	P0BrR186A cloning
	**BrE183R(-)**	GCCAACATTAGCGAGCTCCACAAG	
	**BrV187AF(+)**	GAGCTTCTCCGCGCTATGGTGGC	P0BrV187A cloning
	**BrE183R(-)**	GCCAACATTAGCGAGCTCCACAAG	
	**BrM188AF(+)**	GAGCTTCTCCGCGTTGCGGTGGC	P0BrM188A cloning
	**BrE183R(-)**	GCCAACATTAGCGAGCTCCACAAG	
	**BrV189AF(+)**	GAGCTTCTCCGCGTTATGGCAGC	P0BrV189A cloning
	**BrE183R(-)**	GCCAACATTAGCGAGCTCCACAAG	
	**BrYVAD191AF(+)**	GCTGCAGAGCAATTTCAC	P0BrD191A cloning
	**BrYVAD191AR(-)**	CACCATAACGCGGAGAAGC	
	**BrYVAE192AF(+)**	GCTGATGCACAATTTCAC	P0BrE192A cloning
	**BrYVAD191AR(-)**	CACCATAACGCGGAGAAGC	
	**BrQ193AF(+)**	GCATTTCACAACTCCCGTC	P0BrQ193A cloning
	**BrQ193R(-)**	CTCATCAGCCACCATAACG	
	**BrH195AF(+)**	CAATTTGCAAACTCCCGTC	P0BrH195A cloning
	**BrQ193R(-)**	CTCATCAGCCACCATAACG	
	**BrN196AF(+)**	CAATTTCACGCATCCCGTC	P0BrN196A cloning
	**BrQ193R(-)**	CTCATCAGCCACCATAACG	
	**BrS197AF(+)**	AACGCCCGTCTTCTGTCTCG	P0BrS197A cloning

Experiment	Name	Sequence	Note
	**BrS197R(-)**	GTGAAATTGCTCATCAGCCA	
	**BrR198AF(+)**	CAATTTCACAACTCCGCACTTC	P0^{Br}R198A cloning
	**BrQ193R(-)**	CTCATCAGCCACCATAACG	
	**BrYVAL199AF(+)**	GCACTGTCTCGCCTTGCTG	P0^{Br}L199A cloning
	**BrYVAL199AR(-)**	ACGGGAGTTGTGAAATTGC	
	**BrYVAL200AF(+)**	CTTGCATCTCGCCTTGCTG	P0^{Br}L200A cloning
	**BrYVAL199AR(-)**	ACGGGAGTTGTGAAATTGC	
	**BrS201AF(+)**	AACTCCCGTCTTCTGGCTCGCCTTGCTGTAC	P0^{Br}S201A cloning
	**BrS197R(-)**	GTGAAATTGCTCATCAGCCA	
	**BrR202AF(+)**	GCACTTGCTGTACACTGTTAC	P0^{Br}R202A cloning
	**BrR202R(-)**	AGACAGAAGACGGGAGTTG	
	**BrL203AF(+)**	CGCGCAGCTGTACACTGTTAC	P0^{Br}L203A cloning
	**BrR202R(-)**	AGACAGAAGACGGGAGTTG	
	**BrV205AF(+)**	CGCCTTGCTGCACACTGTTAC	P0^{Br}V205A cloning
	**BrR202R(-)**	AGACAGAAGACGGGAGTTG	
	**BrH206AF(+)**	CGCCTTGCTGTAGCATGTTAC	P0^{Br}H206A cloning
	**BrR202R(-)**	AGACAGAAGACGGGAGTTG	
	**BrYVAC207AF(+)**	CACGCATACAAGATTTATGG	P0^{Br}C207A cloning
	**BrYVAC207AR(-)**	TACAGCAAGGCGAGACAG	
	**BrY208AF(+)**	TGTGCCAAGATTTATGGTGA	P0^{Br}Y208A cloning
	**BrY208R(-)**	GTGTACAGCAAGGCGAGA	
	**BrK209AF(+)**	GCAATTTATGGTGAAGATGGT	P0^{Br}K209A cloning
	**BrK209R(-)**	GTAACAGTGTACAGCAAGGCG	

Experiment	Name	Sequence	Note
	**BrI210AF(+)**	AAGGCATATGGTGAAGATGGT	P0^{Br}I210A cloning
	**BrK209R(-)**	GTAACAGTGTACAGCAAGGCG	
	**BrY211AF(+)**	TGTTACAAGATTGCTGGTGAAGATGGTTTC	P0^{Br}Y211A cloning
	**BrY208R(-)**	GTGTACAGCAAGGCGAGA	
	**BrG212AF(+)**	AAGATTTATGCAGAAGATGGT	P0^{Br}G212A cloning
	**BrK209R(-)**	GTAACAGTGTACAGCAAGGCG	
	**BrE213AF(+)**	AAGATTTATGGTGCAGATGGT	P0^{Br}E213A cloning
	**BrK209R(-)**	GTAACAGTGTACAGCAAGGCG	
	**BrD214AF(+)**	AAGATTTATGGTGAAGCAGGT	P0^{Br}D214A cloning
	**BrK209R(-)**	GTAACAGTGTACAGCAAGGCG	
	**BrYVAG215AF(+)**	GCATTCATTTCTTTTTGG	P0^{Br}G215A cloning
	**BrYVAG215AR(-)**	ATCTTCACCATAAATCTTG	
	**BrF216AF(+)**	GGTGCCATTTCTTTTTGGAGG	P0^{Br}F216A cloning
	**BrF216R(-)**	ATCTTCACCATAAATCTTGTAAC	
	**BrYVAI217AF(+)**	GGTTTCGCATCTTTTTGG	P0^{Br}I217A cloning
	**BrYVAG215AR(-)**	ATCTTCACCATAAATCTTG	
	**BrF219AF(+)**	TCTGCTTGGAGGATTGCCAA	P0^{Br}F219A cloning
	**BrF219R(-)**	AATGAAACCATCTTCACCATAA	
	**BrW220AF(+)**	GCAAGGATTGCCAATCTGG	P0^{Br}W220A cloning
	**BrW220R(-)**	AAAAGAAATGAAACCATCTTCAC	
	**BrR221AF(+)**	TGGGCAATTGCCAATCTGG	P0^{Br}R221A cloning
	**BrW220R(-)**	AAAAGAAATGAAACCATCTTCAC	
	**BrI222AF(+)**	TGGAGGGCAGCCAATCTGG	P0^{Br}I222A cloning

Experiment	Name	Sequence	Note
	**BrW220R(-)**	AAAAGAAATGAAACCATCTTCAC	
	**BrYVAN224AF(+)**	GCACTGGATCATTTCGATTGC	P0^{Br}N224A cloning
	**BrYVAN224AR(-)**	GGCAATCCTCCAAAAAG	
	**BrYVAL225AF(+)**	AATGCAGATCATTTCGATTGC	P0^{Br}L225A cloning
	**BrYVAN224AR(-)**	GGCAATCCTCCAAAAAG	
	**BrYVAD226AF(+)**	AATCTGGCACATTTCGATTGC	P0^{Br}D226A cloning
	**BrYVA N224AR(-)**	GGCAATCCTCCAAAAAG	
	**BrH227AF(+)**	GCATTCGATTGCTTTCTCAC	P0^{Br}H227A cloning
	**BrH227R(-)**	ATCCAGATTGGCAATCCTCC	
	**BrF228AF(+)**	CATGCCGATTGCTTTCTCAC	P0^{Br}F228A cloning
	**BrF228R(-)**	ATCCAGATTGGCAATCCTCC	
	**BrD229AF(+)**	CATTTCGCATGCTTTCTCAC	P0^{Br}D229A cloning
	**BrH227R(-)**	ATCCAGATTGGCAATCCTCC	
	**BrC230AF(+)**	CATTTCGATGCATTTCTCAC	P0^{Br}C230A cloning
	**BrH227R(-)**	ATCCAGATTGGCAATCCTCC	
	**BrF231AF(+)**	TGCGCTCTCACTCCTGAAGAAA	P0^{Br}D231A cloning
	**BrF231R(-)**	ATCGAAATGATCCAGATTGGC	
	**BrYVAL232AF(+)**	GCAACTCCTGAAGAAATCC	P0^{Br}L232A cloning
	**BrYVAL232AR(-)**	AAAGCAATCGAAATGATCC	
	**BrT233AF(+)**	TGCTTTCTCGCTCCTGAAGAAATCC	P0^{Br}T233A cloning
	**BrF231R(-)**	ATCGAAATGATCCAGATTGGC	
	**BrE235AF(+)**	GCAGAAATCCTTTTCAGCTC	P0^{Br}E235A cloning
	**BrE235R(-)**	AGGAGTGAGAAAGCAATCG	

Experiment	Name	Sequence	Note
	**BrE236AF(+)**	GAAGCAATCCTTTTCAGCTC	P0BrE236A cloning
	**BrE235R(-)**	AGGAGTGAGAAAGCAATCG	
	**BrI237AF(+)**	GAAGAAGCACTTTTCAGCTC	P0BrI237A cloning
	**BrE235R(-)**	AGGAGTGAGAAAGCAATCG	
	**BrL238AF(+)**	GAAGAAATCGCATTCAGCTC	P0BrL238A cloning
	**BrE235R(-)**	AGGAGTGAGAAAGCAATCG	
	**BrF239AF(+)**	CTTGCCAGCTCTTCGGTCTAC	P0BrF239A cloning
	**BrF239R(-)**	GATTTCTTCAGGAGTGAGAAAG	
	**BrS240AF(+)**	GCCTCTTCGGTCTACACCGAAATG	P0BrS240A cloning
	**BrS240R(-)**	GAAAAGGATTTCTTCAGGAGTG	
	**BrS241AF(+)**	AGCGCTTCGGTCTACACCGAAATG	P0BrS241A cloning
	**BrS240R(-)**	GAAAAGGATTTCTTCAGGAGTG	
	**BrV243AF(+)**	GCATACACCGAAATGTTTG	P0BrV243A cloning
	**BrV243R(-)**	CGAAGAGCTGAAAAGGATTTC	
	**BrY244AF(+)**	AGCTCTTCGGTCGCCACCGAAATGTTTG	P0BrY244A cloning
	**BrS240R(-)**	GAAAAGGATTTCTTCAGGAGTG	
	**BrT245AF(+)**	AGCTCTTCGGTCTACGCCGAAATGTTTGTA	P0BrT245A cloning
	**BrS240R(-)**	GAAAAGGATTTCTTCAGGAGTG	
	**BrE246AF(+)**	GTCTACACCGCAATGTTTG	P0BrE246A cloning
	**BrV243R(-)**	CGAAGAGCTGAAAAGGATTTC	
	**BrYVAM247AF(+)**	GAAGCATTTGTAGAGCAG	P0BrM247A cloning
	**BrYVAM247AR(-)**	GGTGTAGACCGAAGAGCTG	
	**BrF248AF(+)**	ATGGCTGTAGAGCAGAAGCTG	P0BrF248A cloning

Experiment	Name	Sequence	Note
	**BrF248R(-)**	TTCGGTGTAGACCGAAGAGC	
	**BrYVAV249AF(+)**	GAAATGTTTGCAGAGCAG	$P0^{Br}V249A$ cloning
	**BrYVAM247AR(-)**	GGTGTAGACCGAAGAGCTG	
**Other substitution mutagenesis of $P0^{Br}$**	**BrY61DF(+)**	CGCTCTCTGCTCGATCAGCTTCCTCTCC	$P0^{Br}Y61D$ cloning
	**BrS58R(-)**	AATAAAAATATCAATCTTGAAT	
	**BrA88F(+)**	GAGCTCGCGTTCTGGTTCTCTTTAC	$P0^{Br}A88F$ cloning
	**BrE85R(-)**	TGGTTCAGGGACAAGTATGGAC	
	**BrA97FF(+)**	TCTTTACAAACGGGATATTTCCCC	$P0^{Br}A97F$ cloning
	**BrS91R(-)**	GAACCAGGCGCAGAGCTCTGG	
	**BrA204FF(+)**	CGCCTTTTCGTACACTGTTAC	$P0^{Br}A204F$ cloning
	**BrR202R(-)**	AGACAGAAGACGGGAGTTG	
	**BrA223FF(+)**	TGGAGGATTTTCAATCTGG	$P0^{Br}A223F$ cloning
	**BrW220R(-)**	AAAAGAAATGAAACCATCTTCAC	
**Mutagenesis of consensus-residues in $P0^{Br}$**	**Tu194F(-)**	TCCTTCTCGGAGACCACGTCCAC	$P0^{Br}LP$ (LP63-64AA) and $P0^{Br}LPK$(LPK44A) cloning
	**TuLP63AAR(+)**	GAGCTGCCTGATAGAGCAGAGAG	
	**F219RF(+)**	TCTAGGTGGAGGATTGCCAATC	$P0^{Br}F219R$ cloning
	**F219R(-)**	AATGAAACCATCTTCACC	
	**W220FF(+)**	TCTTTTAGGAGGATTGCCAATC	$P0^{Br}W220F$ cloning
	**F219R(-)**	AATGAAACCATCTTCACC	
**Mutagenesis of BrYV infectious cDNA clone**	**BrpCB301Q2AF(+)**	CATTTGTAGCTCACGACAACTTTCAC	BrYV-$P0^{Q2A}$ cloning
	**BrpCB301Q2AR(+)**	CCATCAACTTAGGATTCCCTCCTGG	
	**BrP0Y61DF(+)**	GATCAGCTTCCTCTCCTTCTCGGAGACC	BrYV-$P0^{Y61D}$ cloning
	**BrP0Y61DR(-)**	GAGCAGAGAGCGAATAAAAATATCAATC	

Experiment	Name	Sequence	Note
	**Br104CCF(+)**	CCTAACGTTTCTTTTAGCAGGTTTATTG	BrYV-P0^{FS} cloning
	**Br104R(-)**	CTTGTCGCGGATGGAGGAATCTAACT	
**Semi quantitative RT-PCR**	**ATG5-F**	AAGCTCATACGCATTCAGGG	*NbATG5* expression level analysis
	**ATG5-R**	GCTTCGGACCTTTGCTACCT	
	**BrP0-sqF**	ATGCAATTTGTAGCTCACGA	BrYV P0 expression level analysis
	**BrP0-sqR**	AGACCGAAGAGCTGAAAAGG	
	**EF1A-F**	CCCCTTCGTCTTCCACTTCA	*NbEF1A* expression level analysis
	**EF1A-R**	GCTTGGTCGGCATCATCTTA	
**Template amplification of probe**	**T7GFP-5**	TAATACGACTCACTATAGGGCGGAAACATCC	GFP Northern blot probe cloning
	**GFP-3-R**	TTATTTGTATAGTTCATCCATGCCATG	
	**BrYV(5101)F**	CCGAGAGCGGACCATAAGGAC	BrYV-A Northern blot probe cloning
	**BrYV(5620)R**	GCGGAGTCGTTCCAGTTTA	

Note: All the sequences are shown in 5'-to-3' direction. Sequences in italics indicate restriction enzyme sites. Primers with mutated sequence are followed by a plus sign (+), and the mutated sequences are underlined. Primers without mutated sequence are followed by a minus sign (-).
